# Supplementary material for: The circadian clock regulates scavenging of fluid-borne substrates by brain border-associated macrophages
Source: bioRxiv. 2025 Dec 11:2025.12.08.693074. Preprint. [Version 1] doi: 10.64898/2025.12.08.693074 (PMC12713129; doi:10.64898/2025.12.08.693074)
Supplement: 1 [file NIHPP2025.12.08.693074v1-supplement-1.pdf]

bioRxiv preprint doi: https://doi.org/10.64898/2025.12.08.693074; this version posted December 11, 2025. The copyright holder for this preprint (which was not certified by peer review) is the author/funder, who has granted bioRxiv a license to display the preprint in perpetuity. It is made available under aCC-BY 4.0 International license.

FIGURE S1

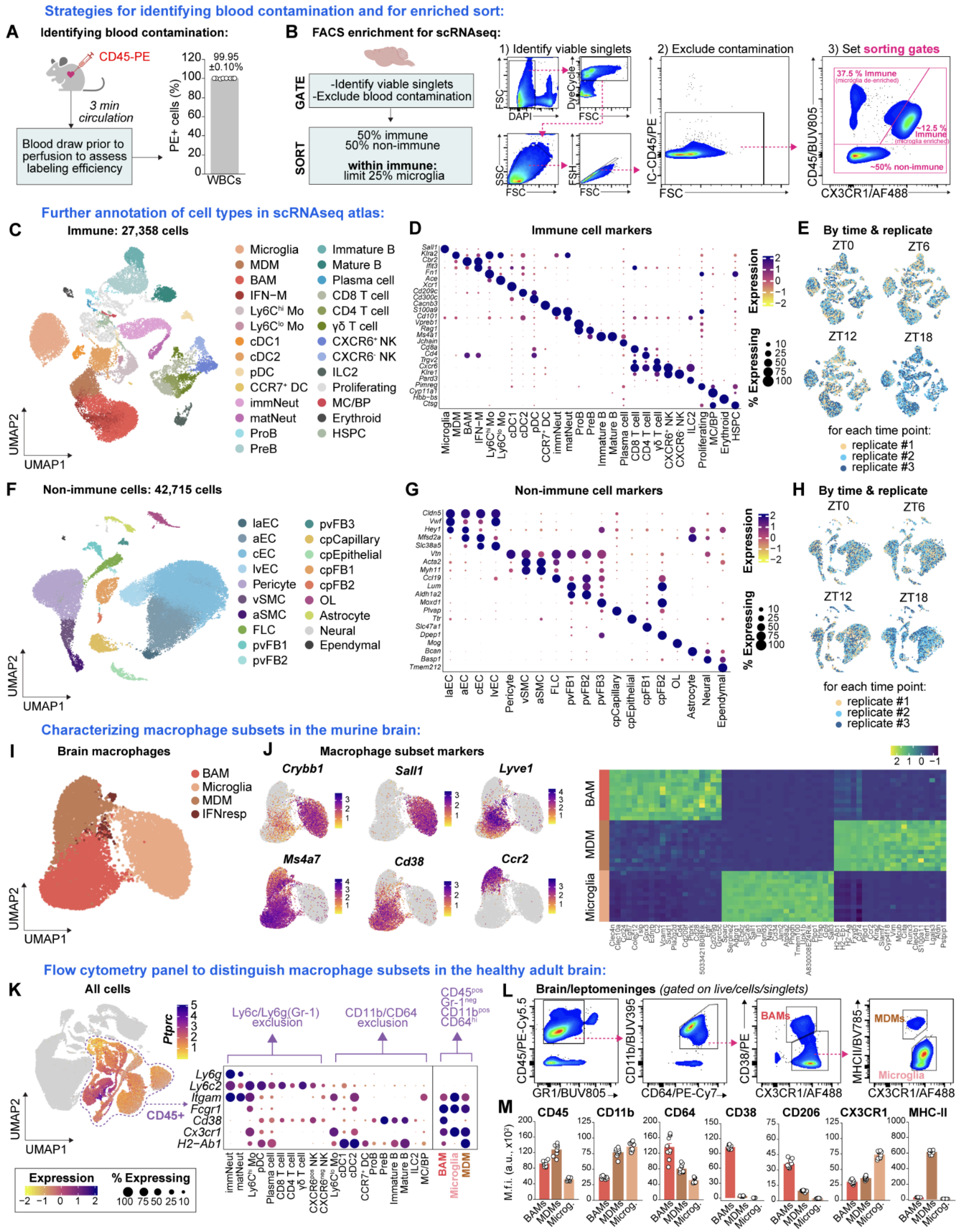

bioRxiv preprint doi: https://doi.org/10.64898/2025.12.08.693074; this version posted December 11, 2025. The copyright holder for this preprint (which was not certified by peer review) is the author/funder, who has granted bioRxiv a license to display the preprint in perpetuity. It is made available under aCC-BY 4.0 International license.

Figure S1. Additional annotation of circadian single-cell atlas and evidence-based flow cytometry panel for macrophage subset distinction in the healthy brain, related to Figure 1

(**A**) Experimental approach for labeling circulating immune cells prior to cardiac perfusion via intracardiac injection of CD45-PE antibody. Following 3 minutes circulation, and prior to cardiac perfusion, blood was acutely sampled to verify white blood cell (WBC) labeling efficacy. Quantification shows mean ± SEM.

(**B**) Gating and sorting strategy prior to single-cell RNA sequencing to de-enrich microglia and enrich other brain-resident immune cells and their niches. Live, viable singlets were dually identified as DAPI^{neg} and Vybrant DyeCycle Ruby^{pos}.

(**C-E**) UMAP of annotated immune cell subsets, heat map of identification genes per cell type, and immune cells split by time and colored by replicate (n of 12 mice, 3 independent samples per time). IFN-M = interferon-responsive macrophages, immNeut and matNeut = immature and mature neutrophils.

(**F-H**) UMAP of annotated non-immune cell subsets, heat map of identification genes per cell type, and non-immune cells split by time and colored by replicate (n of 12 mice, 3 independent samples per time). laEC = large arterial EC, aEC = arterial EC, cEC = capillary EC, lvEC = large venous EC, vSMC = venous SMC, aSMC = arterial SMC, pvFB = perivascular FB, cpFB = choroid plexus FB.

(**I and J**) UMAP of macrophage subsets, feature plot of genes of interest, and heat map of macrophage subset-enriched genes across all 12 independent replicates.

(**K-M**) Feature plot of *Ptprc* expression in whole scRNA-seq atlas, complex heat-map of immune cells demonstrating gene-level expression of markers used for macrophage subset differentiation by flow cytometry, recommended gating strategy, and group-level quantification of recommended surface markers from panel in BAMs, MDMs, and microglia. Points represent individual mice, n of 10 mice.

bioRxiv preprint doi: https://doi.org/10.64898/2025.12.08.693074; this version posted December 11, 2025. The copyright holder for this preprint (which was not certified by peer review) is the author/funder, who has granted bioRxiv a license to display the preprint in perpetuity. It is made available under aCC-BY 4.0 International license.

FIGURE S2

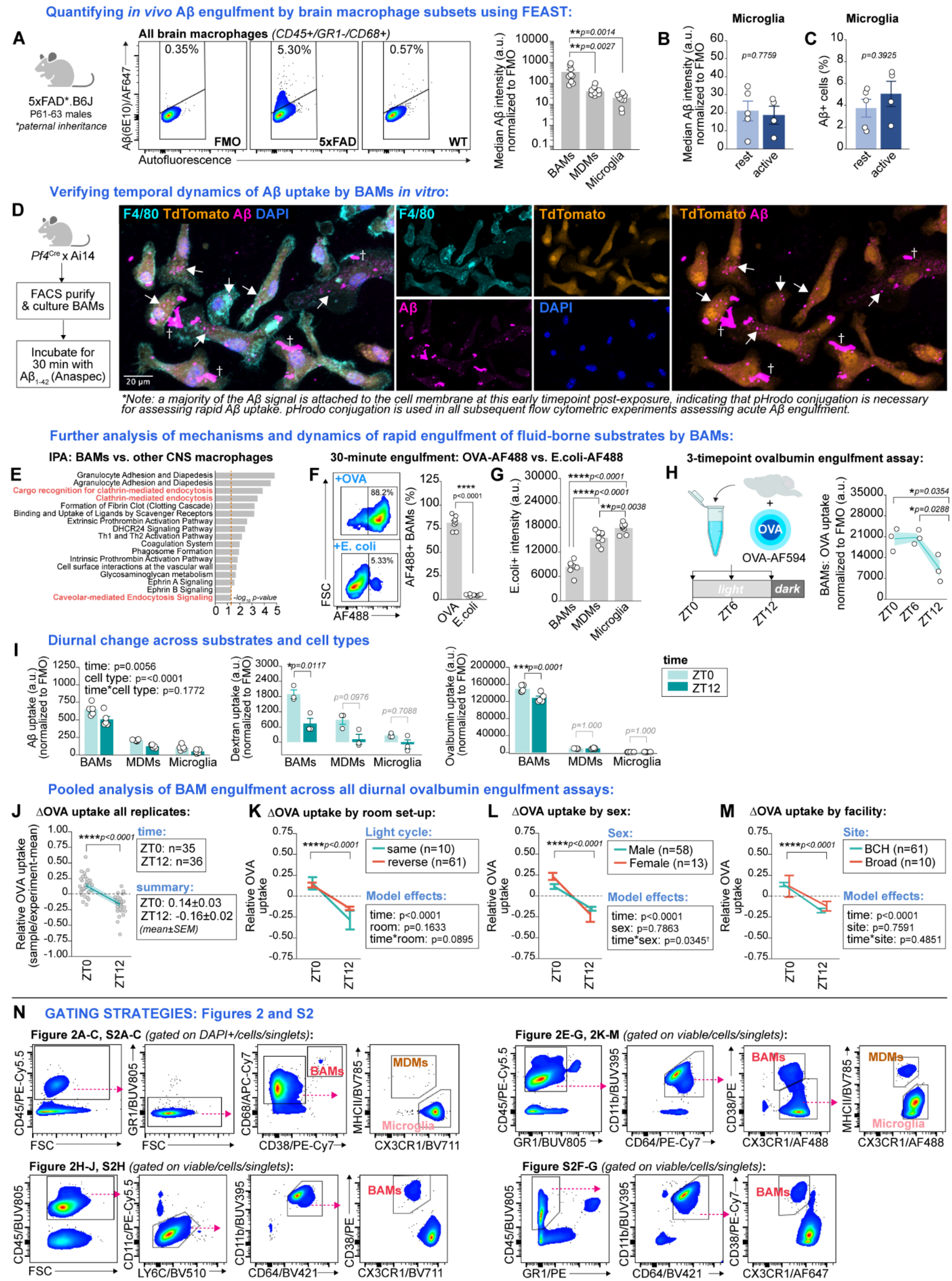

bioRxiv preprint doi: https://doi.org/10.64898/2025.12.08.693074; this version posted December 11, 2025. The copyright holder for this preprint (which was not certified by peer review) is the author/funder, who has granted bioRxiv a license to display the preprint in perpetuity. It is made available under aCC-BY 4.0 International license.

Figure S2. Further characterization of BAM engulfment and its rhythmicity, related to Figure 2

(**A**) Representative pseudocolored plots of Aβ signal in brain macrophages following the FEAST protocol on microdissected cortices from a young 5xFAD male versus a wild-type littermate control, and group-level quantification of Aβ intensity within each macrophage subset. Aβ signal varies by cell type (one-way ANOVA, $F_{2,24}$=10.14, p=0.0006) and is highest in BAMs. N of 9 mice. The P61-63 timepoint is selected to maximize soluble versus insoluble Aβ.

(**B and C**) Quantification of Aβ intensity and Aβ-positivity (%) within microglia, neither of which are significantly different across time at this early time point in amyloidosis. Quantification using two-tailed t-test, n of 4-5 mice per time point.

(**D**) Experimental design and representative image of cultured BAMs following incubation with purified $Aβ_{1-42}$. Aβ is detectable in intracellular vesicular structures within just 30 minutes of exposure (white arrowheads). Importantly, clumps of Aβ also adhered to the cell membrane (denoted by †), highlighting the need for pHrodo conjugation of all Aβ substrates used for measuring acute engulfment capacity.

(**E**) IPA of top 100 genes upregulated in BAMs relative to microglia and MDMs (from cell-level analysis from all time points, n of 12, selected pathways shown). Dashed bar indicates significance threshold.

(**F**) Representative pseudo-colored plots and quantification of substrate positivity in BAMs following 30 minutes digest with either AF488-conjugated ovalbumin or E.coli. Acute capacity for E.coli uptake is far reduced relative to OVA-AF488 uptake (t(7.18)=-29.86, p<0.0001, two-tailed t-test, n of 8).

(**G**) Within E.coli-positive cells, signal varies by cell type ($F_{2,14}$=135.74, p<0.0001). Signal is higher in both MDMs and microglia relative to BAMs (mixed effects model accounting for random variance due to individual sample, n of 8).

(**H**) Experimental design and group-level quantification of OVA-AF594 uptake by BAMs at ZT0, ZT6, and ZT12. Uptake varies by time: $F_{2,6}$=7.90, p=0.0209 (one-way ANOVA, post-hoc testing with Tukey's HSD; n of 3 mice per time point), and is higher at both ZT0 and ZT6 relative to ZT12.

(**I**) Group-level quantification of acute engulfment across cell types and substrates at ZT0 vs. ZT12. While BAMs consistently show a reduction in engulfment at ZT12 vs. ZT0 across substrates, MDMs and microglia do not. Analyzed with time*cell type full factorial mixed effects models accounting for random variance due to individual samples, n of 3-5 per time point).

(**J-M**) Relative OVA uptake by BAMs at ZT0 versus ZT12 across 9 experimental replicates and 71 biological replicates (line and shaded area represent mean and 95% confidence intervals) and relative OVA uptake split by light cycle, sex, and animal facility. Model effects shown are for mixed effects design controlling for random variance due to independent experiments. Sample sizes denote biological replicates. There is a main effect of time on OVA uptake across all conditions. Lines and error bars represent mean and SEM. †No significant post-hoc test for between-sex or within-time comparisons.

(**N**) Flow cytometry gating strategies for data shown in **Figure 2** and **Figure S2.** Note: the staining panel and gating strategy is adjusted for the fixed cell FEAST protocol due to epitope loss following sample preparation.

*For all panels: points represent individual mice, bars and error bars represent mean and SEM. Normalization to background autofluorescence is done per cell type. Illustrations made with BioRender. ****p<0.0001, ***p<0.001, **p<0.01, *p<0.05.*

bioRxiv preprint doi: https://doi.org/10.64898/2025.12.08.693074; this version posted December 11, 2025. The copyright holder for this preprint (which was not certified by peer review) is the author/funder, who has granted bioRxiv a license to display the preprint in perpetuity. It is made available under aCC-BY 4.0 International license.

FIGURE S3

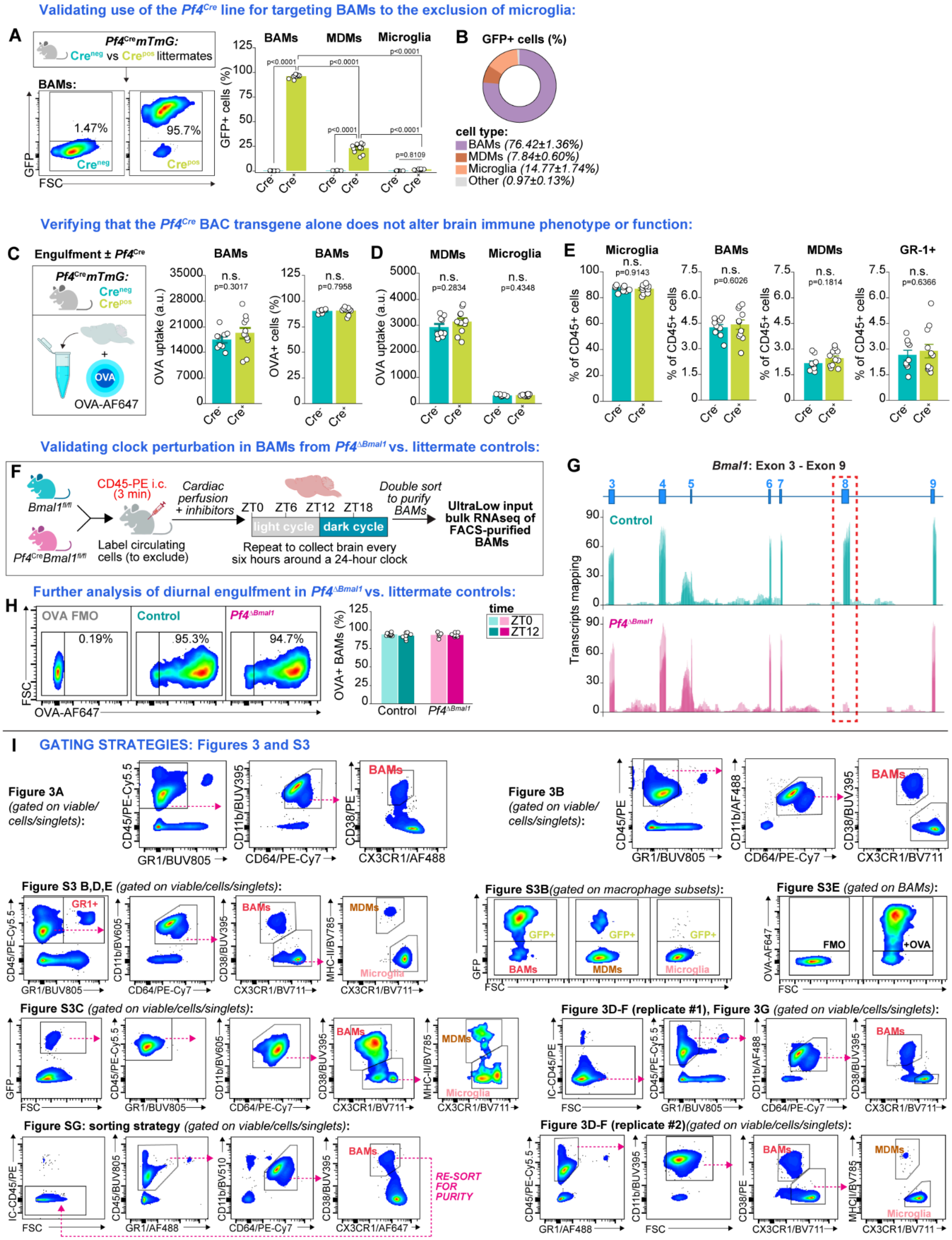

bioRxiv preprint doi: https://doi.org/10.64898/2025.12.08.693074; this version posted December 11, 2025. The copyright holder for this preprint (which was not certified by peer review) is the author/funder, who has granted bioRxiv a license to display the preprint in perpetuity. It is made available under aCC-BY 4.0 International license.

Figure S3. Validation of BAM-targeted circadian disruption, related to Figure 3

(**A**) Pseudo-colored plots and group-level quantification of GFP-positivity across cell types and genotypes in *Pf4*^{Cre}*mTmG* mice. Cell type*genotype interaction: $F_{2,36}$=3211.658, p<0.0001 (mixed effects model accounting for variance due to individual mouse). Selected post-hoc tests shown (all Tukey's HSD; n of 9 Cre^{neg} and 11 Cre^{pos}).

(**B**) Cell type as % of all GFP+ cells in *Pf4*^{Cre}*mTmG* mice. Data shown as average across all Cre^{pos} mice (n of 11).

(**C and D**) Schematic of experimental design, group-level quantification of OVA-AF647 uptake and percent of BAMs positive for OVA-AF647, and group-level quantification of OVA-AF647 uptake by MDMs and microglia. There was no effect of genotype on OVA-AF647 uptake by BAMs, MDMs, or microglia, nor on the % of BAMs positive for OVA-AF647. All comparisons using student's t-test (n of 9 Cre^{neg} and 11 Cre^{pos}).

(**E**) Cell count as % of all CD45+ by genotype. No effect of genotype on microglia, BAM , MDM , or GR-1 cell count (likely monocytes and granulocytes). All comparisons using student's t-test (n of 9 Cre^{neg} and 11 Cre^{pos}).

(**F and G**) Schematic of experimental design for bulk RNA sequencing of BAMs from *Pf4*$^{\Delta Bmal1}$ mice and littermate controls and histogram of reads mapping to the loxP-flanked eighth exon of the *Bmal1* gene at ZT0 in both groups.

(**H**) Pseudo-colored plots and group-level quantification of the percent of BAMs containing detectable OVA-AF647 across time and genotype. No effect of either time nor genotype of OVA-AF647 positivity ($F_{3,33}$=0.6609, p=0.5820; ANOVA; n of 21 control and 16 *Pf4*$^{\Delta Bmal1}$).

(**I**) Flow cytometry gating strategies for **Figure 3** and **Figure S3**.

*For all panels: points represent individual mice, bars and error bars represent mean and SEM. Illustrations made with BioRender. ****p<0.0001, ***p<0.001, **p<0.01, *p<0.05.*

bioRxiv preprint doi: https://doi.org/10.64898/2025.12.08.693074; this version posted December 11, 2025. The copyright holder for this preprint (which was not certified by peer review) is the author/funder, who has granted bioRxiv a license to display the preprint in perpetuity. It is made available under aCC-BY 4.0 International license.

FIGURE S4

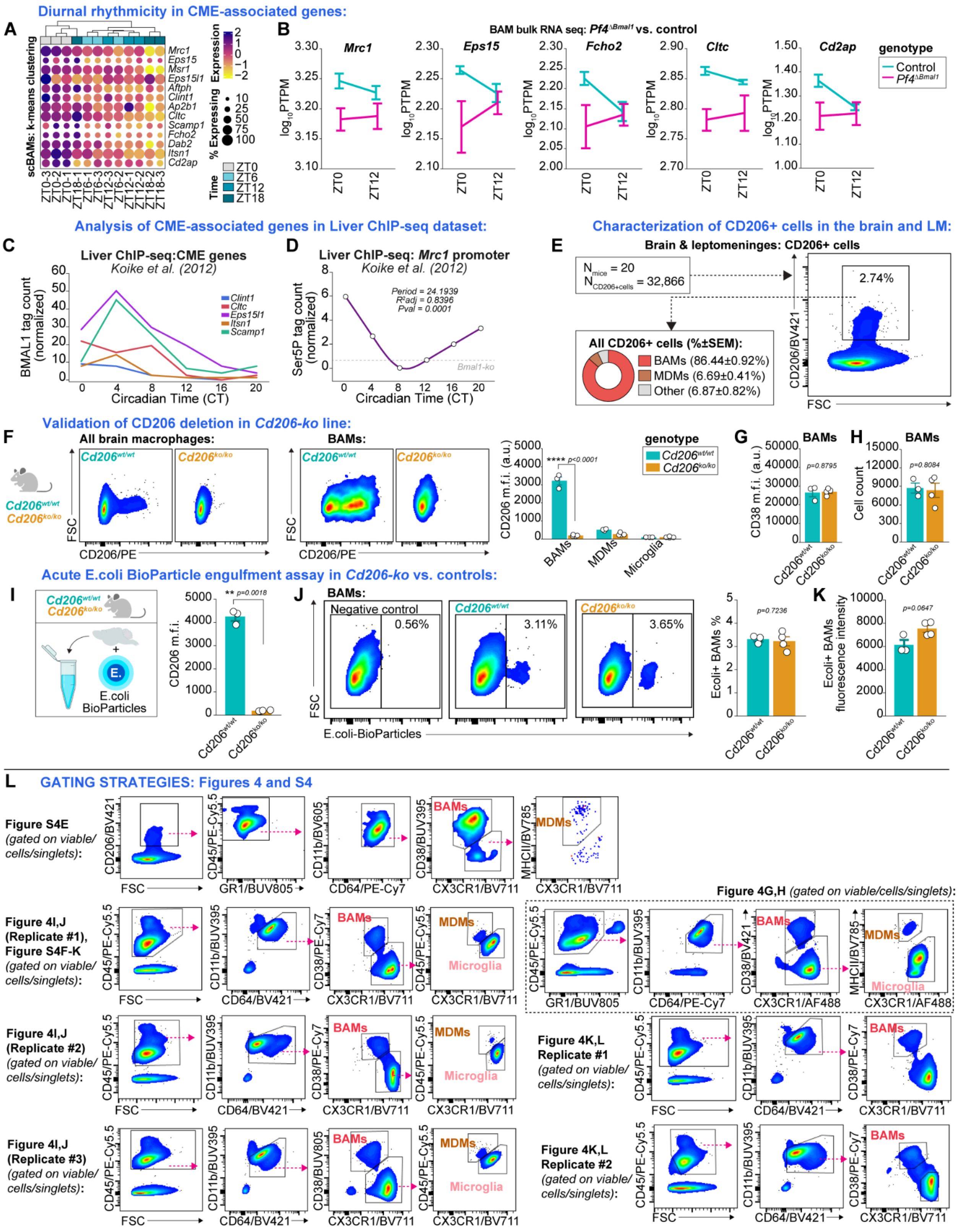

bioRxiv preprint doi: https://doi.org/10.64898/2025.12.08.693074; this version posted December 11, 2025. The copyright holder for this preprint (which was not certified by peer review) is the author/funder, who has granted bioRxiv a license to display the preprint in perpetuity. It is made available under aCC-BY 4.0 International license.

Figure S4. Further exploration of diurnal rhythmicity in CME-associated and validation of CD206 deletion, related to Figure 4.

(**A**) Heatmap of CME-associated gene expression across time and biological replicates in BAMs (n of 3 mice per time point) with unbiased k-means clustering of samples by feature expression.

(**B**) Group-level quantification of $\log_{10}$-transformed pseudo-transcripts per million (PTPM) of CME-associated genes at ZT0 and ZT12 in BAMs from $Pf4^{\Delta Bmal1}$ mice versus their littermate controls.

(**C and D**) Visualization of BMAL1 and Ser5P tagging of genes of interest across time in ChIP-seq dataset of liver (Koike et al., 2012).

(**E**) Psuedo-colored plot of CD206+ cells in brain and quantification of cell type across 20 mice (data re-analyzed from Figure S2.3B-F). BAMs represent 85.59±0.90% of CD206+ cells in the brain (mean ± SEM).

(**F**) Representative pseudo-colored plots and group-level quantification of CD206 expression on brain macrophage subsets from $Cd206^{wt/wt}$ and $Cd206^{ko/ko}$ mice. Cell type*genotype interaction: $F_{2,14}=151.84$, $p<0.0001$ (mixed effects model accounting for individual mouse; post-hoc testing with Tukey's HSD; selected post-hoc tests shown). Points represent individual mice (n of 3 $Cd206^{wt/wt}$ and n of 4 $Cd206^{ko/ko}$).

(**G and H**) Quantification of CD38 expression and BAM yield from $Cd206^{wt/wt}$ and $Cd206^{ko/ko}$ mice. Both CD38 expression (t(2.64)=0.17, p=0.8795) and yield are unaffected by genotype (t(4.90)=0.26, p=0.8084; two-tailed t-tests). Points represent individual mice (n of 3 $Cd206^{wt/wt}$ and n of 4 $Cd206^{ko/ko}$).

(**I-K**) Experimental design, representative pseudo-colored plots, and group-level quantification of E.coli BioParticle uptake in BAMs from $Cd206^{ko/ko}$ mice and their wild-type littermate controls. The knockout was verified by loss of CD206 expression relative to wild-type littermates (t(2.02)=22.93, p=0.0018; two-tailed t-test). Both the proportion of BAMs positive for E.coli BioParticles and the intensity of signal within E.coli+ BAMs were unaffected by genotype (t(4.25)=0.38, p=0.7236 and t(3.69)=2.61, p=0.0647, respectively; two-tailed t-tests). Points represent individual mice (n of 3 $Cd206^{wt/wt}$ and n of 4 $Cd206^{ko/ko}$).

(**L**) Flow cytometry gating strategies for **Figure 4** and **Figure S4**.

For all panels: points represent individual mice, bars and error bars represent mean and SEM. ****p<0.0001, ***p<0.001, **p<0.01, *p<0.05.

bioRxiv preprint doi: https://doi.org/10.64898/2025.12.08.693074; this version posted December 11, 2025. The copyright holder for this preprint (which was not certified by peer review) is the author/funder, who has granted bioRxiv a license to display the preprint in perpetuity. It is made available under aCC-BY 4.0 International license.

FIGURE S5

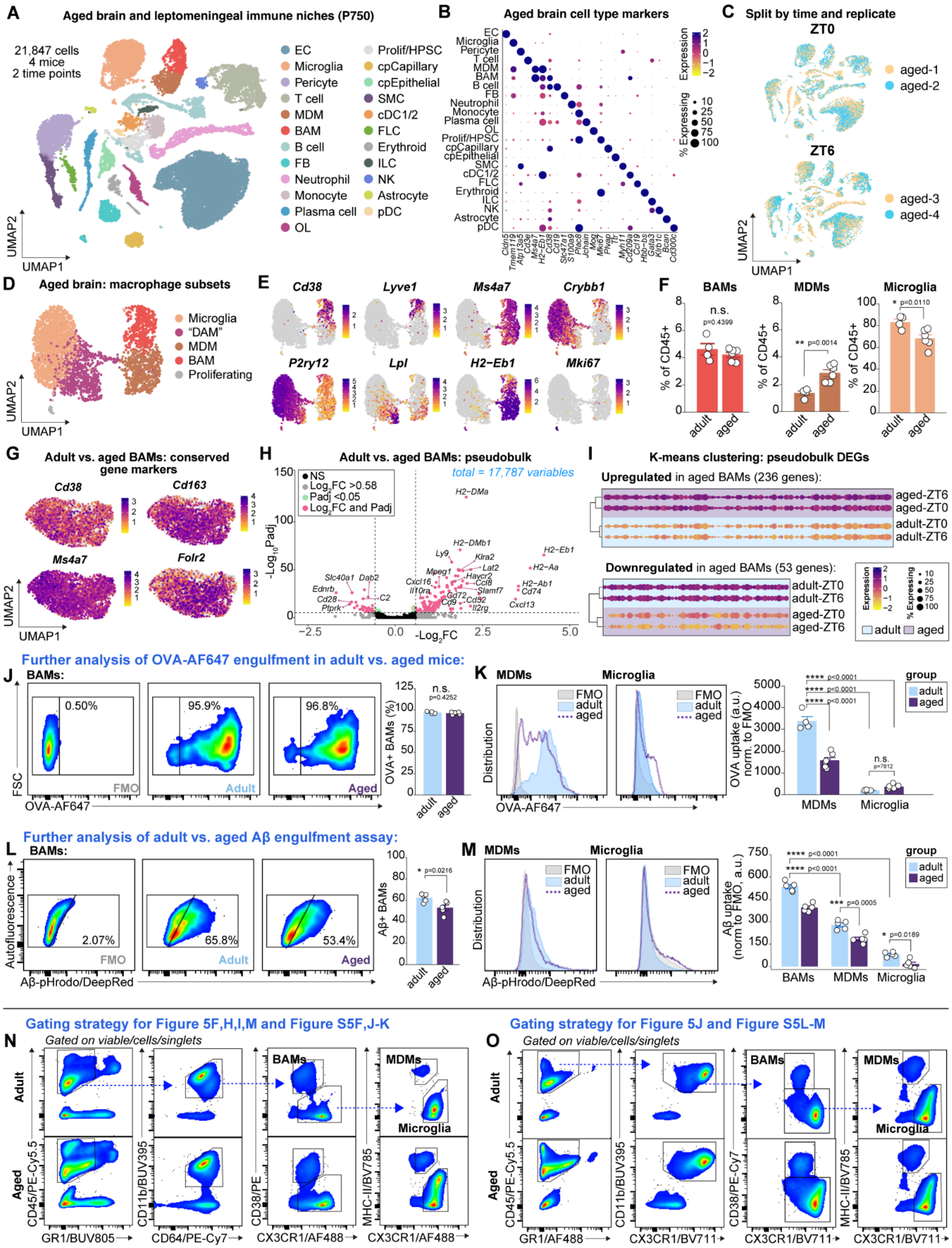

bioRxiv preprint doi: https://doi.org/10.64898/2025.12.08.693074; this version posted December 11, 2025. The copyright holder for this preprint (which was not certified by peer review) is the author/funder, who has granted bioRxiv a license to display the preprint in perpetuity. It is made available under aCC-BY 4.0 International license.

Figure S5. Additional analysis of macrophages subsets in the adult and aged brain, related to Figure 5

(**A-C**) UMAP of annotated cell types, heat map of cell type markers, and dataset split by time and replicate (n = 2 independent samples per time point).

(**D and E**) UMAP of macrophage subsets and feature plots of identification genes per subset (from aged brain dataset; 3,573 macrophages). DAM = "disease-associated microglia".

(**F**) BAM, MDM, and microglia counts by flow cytometry, shown as % of CD45-positive cells in the brain and leptomeninges. BAM counts are unchanged by age ($t(4.17)=-0.85$, $p=0.4399$), but MDMs are increased ($t(7.81)=4.84$, $p=0.0014$) and microglia are decreased with age ($t(7.84)=-3.31$, $p=0.0110$; two-tailed t-tests). Points represent individual mice, n = 4 adult and 6 aged mice, all male B6N.

(**G**) Feature plot of conserved genetic markers of BAM identity across age.

(**H**) Volcano plot comparing BAMs from adult vs. aged mice via pseudobulk analysis; n = 10 total independent samples (6 adult, 4 aged, both ZT0 and ZT6 from each age included in comparison, all samples obtained in same experiment/preparation). FC threshold set to 0.58 (equal to a 1.5-fold change in expression).

(**I**) K-mean clustering of DEGs from pseudobulk analysis, split by pattern of expression. For both up- and down-regulated genes, unbiased clustering still distinguishes adult from aged BAMs across ZT0 and ZT6.

(**J**) Representative pseudo-colored plots and group-level comparison of the proportion of BAMs containing detectable levels of OVA-AF647 from adult vs. aged OVA engulfment assay (**Figure 2.5I**). The percent of OVA+ BAMs is unchanged by age ($t(6.76)=-0.85$, $p=0.4252$; two-tailed t-test with independent samples). Points represent individual mice, n = 4 adult and 6 aged mice, all male B6N collected at ZT0.

(**K**) Representative histograms and group-level quantification of OVA-AF647 uptake in MDMs and microglia from adult vs. aged mice: age*cell type interaction ($F(3,16)=129.60$, $p<0.0001$; mixed effects model with random variable of individual mouse). Uptake was reduced in aged MDMs relative to adult MDMs ($p<0.0001$). Regardless of age, MDMs surpassed microglia in uptake (all $p<0.0001$). Microglia engulfment was unaffected by age ($p=0.7812$).

(**L**) Representative pseudo-colored plots and group-level comparison of the proportion of BAMs containing detectable levels of Aβ-pHrodo from adult vs. aged Aβ engulfment assay. The percent of BAMs with detectable Aβ over background is reduced in aged BAMs ($t(6.89)=-2.96$, $p=0.0216$; two-tailed t-test with independent samples). Points represent individual mice, n = 5 mice per age.

(**M**) Representative histograms and group-level quantification of Aβ uptake across cell types and ages. Age*cell interaction: $F(2,16)=8.99$, $p=0.0024$ (mixed effects model with random variable of individual mice, n = 5 mice per age).

(**N and O**) Gating strategies for **Figure 5** and **Figure S5**. The same gating strategy was applied to adult and aged samples from each experiment and was compatible with the increased autofluorescence and leukocyte infiltration present in the aged brain.

*For all panels: points represent individual mice, bars and error bars represent mean and SEM. FMOs were generated per age to account for differences in background autofluorescence. Post-hoc testing with Tukey's HSD. Illustrations made with BioRender. ****p<0.0001, ***p<0.001, **p<0.01, *p<0.05.*

bioRxiv preprint doi: https://doi.org/10.64898/2025.12.08.693074; this version posted December 11, 2025. The copyright holder for this preprint (which was not certified by peer review) is the author/funder, who has granted bioRxiv a license to display the preprint in perpetuity. It is made available under aCC-BY 4.0 International license.

FIGURE S6

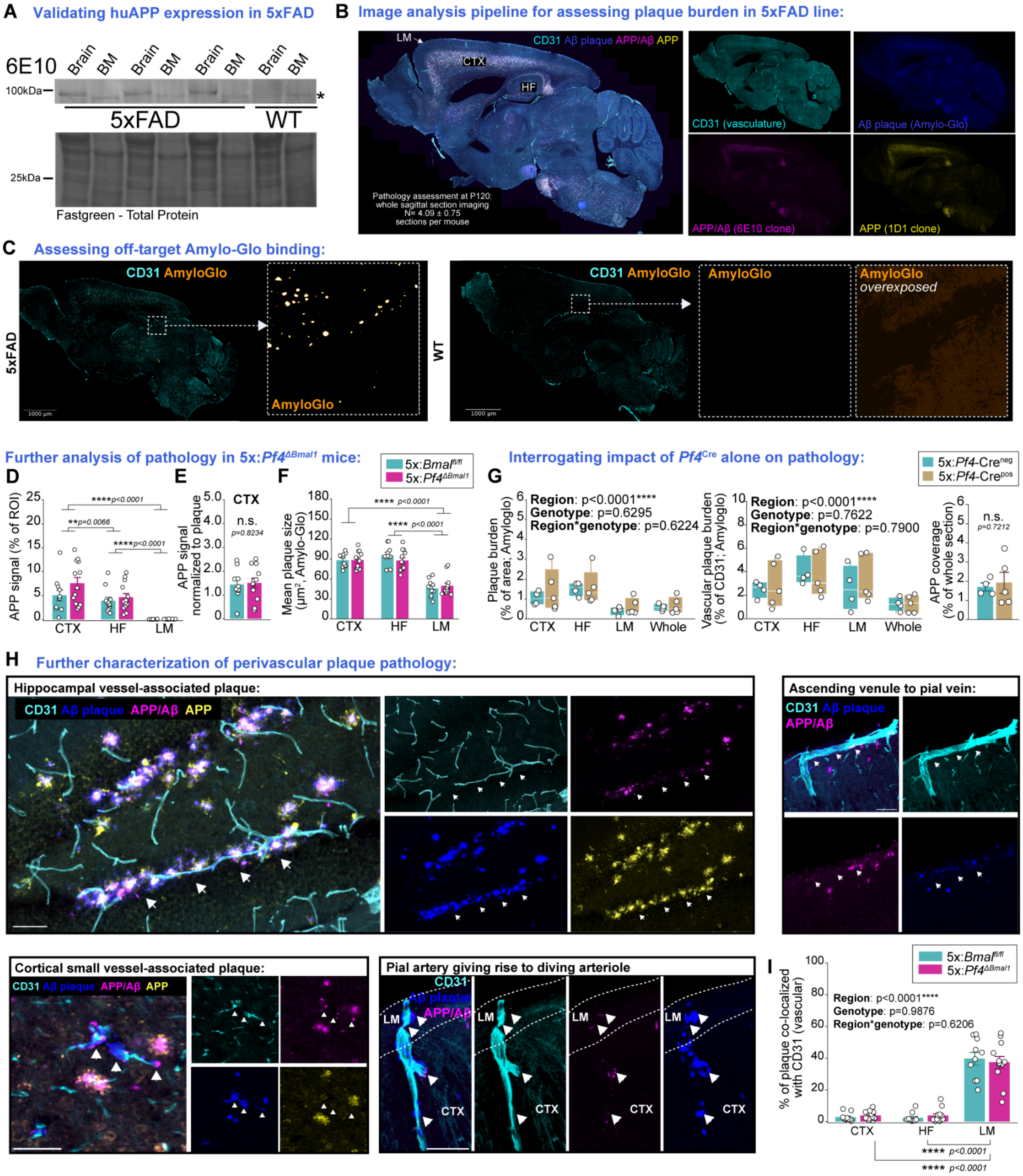

bioRxiv preprint doi: https://doi.org/10.64898/2025.12.08.693074; this version posted December 11, 2025. The copyright holder for this preprint (which was not certified by peer review) is the author/funder, who has granted bioRxiv a license to display the preprint in perpetuity. It is made available under aCC-BY 4.0 International license.

Figure S6. Further characterization of brain border amyloid pathology, related to Figure 6.

(**A**) Human APP expression across brain and bone marrow (BM) of n of 3 5xFAD mice and a wild-type littermate control. Full-length human APP is only detected in the brain, but not bone marrow, of 5xFAD samples, and is not detected in the wild-type. *Denotes non-specific band.

(**B**) Representative sagittal section slide-scanner microscopy image from a 5xFAD brain showing anatomical regions segmented for further analysis: the dorsal leptomeninges (LM), cortex (CTX), and hippocampal formation (HF).

(**C**) Representative whole-section images of Amylo-Glo staining in 5xFAD versus wild-type mouse. No signal is detected in the wild-type mouse.

(**D**) Quantification of APP signal across regions and genotypes. Very little APP is detectable above background in the leptomeninges. While there was a main effect of region on APP signal (p<0.0001), there was no effect of genotype (p=0.2419) nor region*genotype interaction effect (p=0.1373). Quantified using mixed effects model controlling for variance due to individual mouse and sex; post-hoc testing with Tukey's HSD. N of 10-12 per genotype.

(**E**) Cortical APP signal normalized to cortical plaque burden in 5x:*Pf4*$^{\Delta Bmal1}$ mice versus their littermate controls. Quantified with two-tailed t-test, n of 10-12 per genotype.

(**F**) Group-level quantification of plaque size per genotype and region. Main effect of region (p<0.0001), but not genotype (p=0.7599), and no region*genotype interaction (p=0.2825) on plaque size. Quantified using mixed effects model controlling for variance due to individual mouse and sex; post-hoc testing with Tukey's HSD. N of 10-12 per genotype.

(**G**) Group-level quantification of amyloid pathology in 5xFAD mice expressing *Pf4*^{Cre} (5x:*Pf4*^{Cre}) versus their Cre-negative littermates (5xFAD). CTX = cortex, HF = hippocampal formation, LM = leptomeninges. No effect of genotype on plaque burden or vascular plaque burden (mixed effects models controlling for mouse and sex), nor on APP coverage (two-tailed student's t-test). N of 4 to 5 per genotype.

(**H**) Further representative confocal microscopy images of perivascular plaque hippocampal small vessels, cortical small vessels, and diving pial arteries. Perivascular plaques are indicated via white arrowheads. Scale bars = 50μm.

(**I**) Group-level quantification of plaque colocalized with CD31 per genotype and region. Main effect of region, but not genotype, on likelihood of plaque to be vessel-associated (quantified using mixed effects model controlling for variance due to individual mouse and sex; post-hoc testing with Tukey's HSD). N of 10-12 per genotype.

*For all panels: points represent individual mice, bars and error bars represent mean and SEM, box-and-whisker plots represent median and IQR (points unconnected to whiskers are considered outliers by IQR). ****p<0.0001, ***p<0.001, **p<0.01, *p<0.05.*

bioRxiv preprint doi: https://doi.org/10.64898/2025.12.08.693074; this version posted December 11, 2025. The copyright holder for this preprint (which was not certified by peer review) is the author/funder, who has granted bioRxiv a license to display the preprint in perpetuity. It is made available under aCC-BY 4.0 International license.

Table S1:

p_val	avg_log2FC	pct.1	pct.2	p_val_adj	cluster	gene
0	9.68961233	0.996	0.006	0	EC	Cldn5
0	9.50081725	0.991	0.011	0	EC	Slco1a4
0	9.608165	0.979	0.01	0	EC	Spock2
0	8.03854753	0.995	0.072	0	EC	Slc2a1
0	10.3167476	0.802	0.002	0	EC	Degs2
0	8.87198231	0.804	0.006	0	EC	Foxq1
0	8.26345137	0.774	0.011	0	EC	Ocln
0	9.94392611	0.741	0.002	0	EC	Srarp
0	8.11202698	0.707	0.013	0	EC	Prom1
0	8.16221795	0.674	0.006	0	EC	AU021092
0	8.09879945	0.614	0.009	0	EC	Slc16a4
0	9.44577294	0.558	0.002	0	EC	Slc1a1
0	7.99742523	0.48	0.005	0	EC	Slc38a5
0	8.72122408	0.443	0.004	0	EC	Zic3
0	8.41976954	0.391	0.003	0	EC	Sgpp2
0	10.3277778	0.38	0.001	0	EC	Slc19a3
0	9.87952059	0.349	0.001	0	EC	Tmem252
0	8.36790358	0.349	0.002	0	EC	Tbx1
0	7.78099883	0.323	0.008	0	EC	Vwf
0	7.99233633	0.294	0.003	0	EC	Edn1
0	6.50983711	0.993	0.022	0	Pericyte	Atp13a5
0	5.79065024	1	0.048	0	Pericyte	Vtn
0	6.20503394	0.988	0.037	0	Pericyte	Kcnj8
0	6.17711203	0.996	0.054	0	Pericyte	Sod3
0	5.78627791	0.973	0.041	0	Pericyte	P2ry14
0	5.87563706	0.945	0.03	0	Pericyte	Art3
0	6.55160307	0.92	0.023	0	Pericyte	Abcc9
0	5.7185079	0.862	0.034	0	Pericyte	S1pr3
0	5.75900119	0.869	0.041	0	Pericyte	Pde8b
0	5.79027321	0.833	0.053	0	Pericyte	Uchl1
0	7.1487332	0.722	0.01	0	Pericyte	Ggt1
0	6.27125826	0.68	0.014	0	Pericyte	Pla1a
0	5.84879296	0.66	0.017	0	Pericyte	Trpc3
0	8.0465329	0.616	0.005	0	Pericyte	Nodal
0	5.82931717	0.438	0.01	0	Pericyte	Ajap1
0	6.87910466	0.306	0.004	0	Pericyte	Nxph4
0	8.68939104	0.288	0.001	0	Pericyte	Saa1
0	5.69106296	0.293	0.008	0	Pericyte	Galnt17
0	8.35205073	0.266	0.001	0	Pericyte	Sfrp2
0	8.60062729	0.25	0.001	0	Pericyte	Saa2
0	7.56436391	0.996	0.04	0	Microglia	Tmem119
0	7.77153246	0.999	0.085	0	Microglia	P2ry12
0	6.77058324	0.824	0.021	0	Microglia	Lag3
0	10.9285832	0.77	0.002	0	Microglia	Slc2a5
0	6.78752299	0.774	0.015	0	Microglia	Sall1
0	8.47086362	0.721	0.006	0	Microglia	Gal3st4
0	7.48161847	0.651	0.008	0	Microglia	Mlxipl
0	7.88129815	0.629	0.01	0	Microglia	Adora3
0	8.31163781	0.607	0.005	0	Microglia	Csmd3
0	8.16356773	0.58	0.005	0	Microglia	Capn3
0	7.1872281	0.574	0.007	0	Microglia	Nav3
0	7.74739257	0.528	0.006	0	Microglia	Lrrc3
0	8.42778772	0.473	0.004	0	Microglia	Gm2629
0	7.52847025	0.405	0.007	0	Microglia	Gpr84
0	9.80936698	0.35	0.001	0	Microglia	Upk1b
0	7.54977624	0.346	0.004	0	Microglia	A830008E24Rik
0	8.71709868	0.312	0.002	0	Microglia	Gp9
0	8.54511983	0.292	0.002	0	Microglia	Il1a
0	7.04341611	0.277	0.003	0	Microglia	Sall3
0	7.09265658	0.251	0.004	0	Microglia	Gm33699
0	6.63068134	0.994	0.031	0	BAM	Pf4
0	6.90908212	0.969	0.016	0	BAM	Cbr2
0	6.57241932	0.999	0.056	0	BAM	Mrc1

bioRxiv preprint doi: https://doi.org/10.64898/2025.12.08.693074; this version posted December 11, 2025. The copyright holder for this preprint (which was not certified by peer review) is the author/funder, who has granted bioRxiv a license to display the preprint in perpetuity. It is made available under aCC-BY 4.0 International license.

0	6.39377622	0.943	0.028	0	BAM	Ms4a4a
0	6.64557912	0.866	0.016	0	BAM	Igf1
0	7.80103942	0.843	0.01	0	BAM	Clec4n
0	6.92076751	0.79	0.018	0	BAM	Cd163
0	7.98713059	0.689	0.005	0	BAM	Ccl24
0	7.07431862	0.68	0.006	0	BAM	Folr2
0	6.80736996	0.627	0.012	0	BAM	Gpx3
0	7.15198448	0.602	0.004	0	BAM	Lyve1
0	6.10234976	0.566	0.013	0	BAM	C2
0	6.41781473	0.489	0.009	0	BAM	C4b
0	6.59321985	0.455	0.005	0	BAM	Ccl7
0	8.00191903	0.413	0.002	0	BAM	Pla2g2d
0	10.2679162	0.349	0.001	0	BAM	Cd209f
0	6.26120937	0.329	0.005	0	BAM	Ms4a14
0	6.94990206	0.301	0.003	0	BAM	Ccl8
0	7.12726412	0.289	0.003	0	BAM	5033421B08Rik
0	10.1139306	0.255	0.001	0	BAM	Cd209g
0	5.08691282	0.942	0.064	0	MDM	H2-Eb1
0	4.88496124	0.945	0.076	0	MDM	H2-Ab1
0	4.81673775	0.944	0.076	0	MDM	H2-Aa
0	4.56976256	0.973	0.127	0	MDM	Cd74
0	4.20787103	0.94	0.139	0	MDM	H2-DMb1
0	3.56448093	0.946	0.158	0	MDM	H2-DMa
0	4.15207582	0.853	0.094	0	MDM	Cxcl16
0	3.45491386	0.925	0.178	0	MDM	Apobec1
0	4.05915004	0.776	0.08	0	MDM	Slamf9
0	4.48943157	0.613	0.041	0	MDM	Lilra5
0	4.80033877	0.558	0.025	0	MDM	Klra2
0	3.49668095	0.563	0.07	0	MDM	Gpr65
0	4.18076057	0.473	0.033	0	MDM	Scimp
0	3.92204624	0.438	0.033	0	MDM	Rab7b
0	4.15661429	0.427	0.026	0	MDM	Clec4b1
0	3.68946782	0.4	0.037	0	MDM	Tnfsf13b
0	3.95045348	0.287	0.021	0	MDM	Fcgr4
0	3.51818673	0.288	0.026	0	MDM	Creb5
0	4.36237525	0.261	0.016	0	MDM	Gm35154
0	3.70550599	0.253	0.02	0	MDM	Sirpb1a
0	8.22438741	0.913	0.003	0	T cell	Cd3e
0	6.16199591	0.928	0.028	0	T cell	Ms4a4b
0	7.18150016	0.903	0.008	0	T cell	Cd3g
0	6.18202153	0.909	0.025	0	T cell	Lck
0	6.20939865	0.902	0.025	0	T cell	Thy1
0	8.17024028	0.876	0.006	0	T cell	Cd3d
0	6.58987468	0.873	0.021	0	T cell	Lat
0	6.34192567	0.784	0.018	0	T cell	Itk
0	6.3714085	0.672	0.011	0	T cell	Cd247
0	6.52971199	0.616	0.011	0	T cell	Gm2682
0	6.49368805	0.593	0.011	0	T cell	Trac
0	8.54906305	0.509	0.003	0	T cell	Cd5
0	6.68731209	0.503	0.007	0	T cell	Bcl11b
0	9.99670905	0.449	0.001	0	T cell	Cd6
0	9.58856478	0.438	0.001	0	T cell	Themis
0	6.21790947	0.441	0.006	0	T cell	Sh2d1a
0	9.95218123	0.416	0.001	0	T cell	Cd8a
0	8.95045556	0.413	0.002	0	T cell	Cd8b1
0	6.38179549	0.295	0.006	0	T cell	Camk4
0	9.84292727	0.262	0.001	0	T cell	Gzmk
0	11.3732699	0.999	0.002	0	B cell	Cd79a
0	11.6027509	0.956	0.002	0	B cell	Cd19
0	9.1603041	0.911	0.007	0	B cell	Spib
0	11.6152113	0.88	0.001	0	B cell	Vpreb3
0	9.17138942	0.85	0.004	0	B cell	Pou2af1
0	8.48173086	0.852	0.008	0	B cell	Fcrla
0	9.48823305	0.715	0.005	0	B cell	Fam129c
0	8.6884198	0.709	0.011	0	B cell	Tnfrsf13c
0	10.9968871	0.654	0.001	0	B cell	Pax5
0	11.7392847	0.553	0.001	0	B cell	Fcmr

bioRxiv preprint doi: https://doi.org/10.64898/2025.12.08.693074; this version posted December 11, 2025. The copyright holder for this preprint (which was not certified by peer review) is the author/funder, who has granted bioRxiv a license to display the preprint in perpetuity. It is made available under aCC-BY 4.0 International license.

0	12.3617804	0.541	0.001	0	B cell	Ms4a1
0	9.96909337	0.535	0.001	0	B cell	Gm30211
0	9.02692436	0.391	0.002	0	B cell	Gm37065
0	12.9307894	0.367	0	0	B cell	4930426D05Rik
0	12.2279554	0.364	0	0	B cell	Rag1
0	8.75976774	0.314	0.002	0	B cell	Slc12a3
0	11.071357	0.3	0.001	0	B cell	Gm34095
0	10.2259199	0.262	0.001	0	B cell	Iglv3
0	11.5986202	0.258	0	0	B cell	Klhl14
0	10.5564502	0.25	0.001	0	B cell	2010309G21Rik
0	4.9256124	0.939	0.071	0	cDC1/2	Olfm1
0	6.60651372	0.785	0.02	0	cDC1/2	Cd209a
0	5.254021	0.743	0.027	0	cDC1/2	Clec4b1
0	5.7731489	0.695	0.013	0	cDC1/2	Flt3
0	5.85296459	0.64	0.011	0	cDC1/2	Hepacam2
0	7.43766354	0.613	0.006	0	cDC1/2	Cd209c
0	5.71713141	0.592	0.012	0	cDC1/2	Kctd14
0	6.71881318	0.516	0.005	0	cDC1/2	Tnip3
0	6.36548471	0.501	0.007	0	cDC1/2	Kcne3
0	5.75415382	0.477	0.008	0	cDC1/2	Dpep2
0	5.75313629	0.452	0.008	0	cDC1/2	Skint3
0	5.69624125	0.433	0.016	0	cDC1/2	Ear2
0	7.52043878	0.419	0.003	0	cDC1/2	Nccrp1
0	5.52381999	0.363	0.012	0	cDC1/2	Cldn1
0	8.38892862	0.328	0.001	0	cDC1/2	Ffar4
0	5.49895552	0.312	0.005	0	cDC1/2	Ryr1
0	5.64067325	0.3	0.004	0	cDC1/2	Ccdc170
0	6.48838061	0.274	0.006	0	cDC1/2	Clec9a
0	4.97326292	0.268	0.006	0	cDC1/2	Gm15156
0	4.81534315	0.261	0.006	0	cDC1/2	F2rl2
0	12.8212055	0.996	0.001	0	Neutrophil	S100a9
0	12.5279033	0.996	0.003	0	Neutrophil	S100a8
0	12.2602884	0.945	0.001	0	Neutrophil	Wfdc21
0	11.0051824	0.921	0.006	0	Neutrophil	Lcn2
0	12.6849213	0.823	0.001	0	Neutrophil	Retnlg
0	14.0070932	0.801	0	0	Neutrophil	Ngp
0	14.5295695	0.79	0	0	Neutrophil	Ly6g
0	13.7536648	0.761	0	0	Neutrophil	Camp
0	13.1717427	0.703	0	0	Neutrophil	Ltf
0	13.3753477	0.67	0	0	Neutrophil	Itgb2l
0	14.2854677	0.523	0	0	Neutrophil	Ankrd22
0	11.4234734	0.504	0.001	0	Neutrophil	9830107B12Rik
0	11.7314991	0.442	0	0	Neutrophil	Il1f9
0	12.4360568	0.434	0	0	Neutrophil	4930438A08Rik
0	12.0384887	0.402	0	0	Neutrophil	Ceacam10
0	12.2367177	0.388	0	0	Neutrophil	Slco4c1
0	11.1207388	0.308	0	0	Neutrophil	Lin28a
0	11.0706479	0.304	0	0	Neutrophil	Mrgpra2b
0	11.4554211	0.281	0	0	Neutrophil	Mirt2
0	12.6149909	0.258	0	0	Neutrophil	Orm1
0	6.12617137	0.916	0.069	0	SMC	Tpm2
0	7.91475259	0.831	0.026	0	SMC	Myh11
0	8.89543398	0.815	0.019	0	SMC	Acta2
0	6.0100523	0.797	0.04	0	SMC	Mustn1
0	8.45119203	0.757	0.005	0	SMC	Map3k7cl
0	7.74055888	0.732	0.006	0	SMC	Nrip2
0	8.05873068	0.728	0.018	0	SMC	Tagln
0	8.76216654	0.534	0.002	0	SMC	Gpr20
0	9.53124303	0.519	0.001	0	SMC	Olfr558
0	6.77017092	0.49	0.008	0	SMC	Lmod1
0	5.65964756	0.505	0.028	0	SMC	Sncg
0	9.59270863	0.467	0.002	0	SMC	Pdlim3
0	6.70231311	0.409	0.005	0	SMC	Dhx58os
0	5.92643236	0.419	0.015	0	SMC	Myom1
0	10.8308575	0.371	0.001	0	SMC	Pln
0	6.5383591	0.392	0.023	0	SMC	Palld
0	5.7517106	0.371	0.011	0	SMC	Fgf1

bioRxiv preprint doi: https://doi.org/10.64898/2025.12.08.693074; this version posted December 11, 2025. The copyright holder for this preprint (which was not certified by peer review) is the author/funder, who has granted bioRxiv a license to display the preprint in perpetuity. It is made available under aCC-BY 4.0 International license.

0	6.76881751	0.293	0.003	0	SMC	Jph2
0	6.77372395	0.278	0.004	0	SMC	Ctnna3
0	6.9361611	0.265	0.005	0	SMC	Kcnab1
0	13.4119433	0.992	0.002	0	cpCapillary	Plvap
0	9.56564909	0.929	0.005	0	cpCapillary	Exoc3l2
0	7.81515649	0.928	0.009	0	cpCapillary	Col13a1
0	8.04680923	0.937	0.024	0	cpCapillary	Igfbp3
0	7.48668716	0.937	0.034	0	cpCapillary	Cd300lg
0	7.33102167	0.904	0.036	0	cpCapillary	Slc43a3
0	6.99953258	0.889	0.026	0	cpCapillary	Fam167b
0	9.98951381	0.812	0.003	0	cpCapillary	Car8
0	6.72577652	0.79	0.017	0	cpCapillary	Sema3f
0	12.4692354	0.701	0.001	0	cpCapillary	Esm1
0	11.2615748	0.688	0	0	cpCapillary	Ces2e
0	14.1622199	0.661	0	0	cpCapillary	Gpihbp1
0	17.6573119	0.656	0	0	cpCapillary	Rbp7
0	10.5796158	0.645	0.003	0	cpCapillary	Btnl9
0	8.04110701	0.618	0.004	0	cpCapillary	Ntf3
0	7.00538155	0.57	0.007	0	cpCapillary	Piezo2
0	12.3810452	0.499	0	0	cpCapillary	Olfr1396
0	7.09372627	0.425	0.005	0	cpCapillary	Cyp26b1
0	7.39222567	0.389	0.003	0	cpCapillary	Enpp6
0	10.1213472	0.262	0	0	cpCapillary	Lrrc3b
0	7.72244916	0.962	0.007	0	NK cell	Klrb1c
0	8.70162964	0.958	0.003	0	NK cell	Ncr1
0	6.64225028	0.946	0.021	0	NK cell	Cd7
0	7.13283868	0.928	0.018	0	NK cell	Xcl1
0	7.36014105	0.847	0.007	0	NK cell	Klre1
0	6.91410016	0.8	0.013	0	NK cell	Klrb1f
0	6.73713784	0.672	0.006	0	NK cell	Klrc2
0	6.46224238	0.674	0.01	0	NK cell	Prf1
0	6.44897557	0.658	0.018	0	NK cell	Klrb1b
0	6.66529284	0.63	0.011	0	NK cell	Gm36723
0	7.58447283	0.583	0.002	0	NK cell	Styk1
0	8.66394311	0.53	0.002	0	NK cell	Klrb1a
0	7.47721397	0.479	0.005	0	NK cell	Gzmb
0	7.76077336	0.443	0.002	0	NK cell	Klra9
0	6.78447658	0.425	0.004	0	NK cell	Tnfrsf9
0	8.33757356	0.363	0.002	0	NK cell	Klri2
0	9.15891232	0.363	0.002	0	NK cell	Gzma
0	8.88152703	0.339	0.001	0	NK cell	Klra8
0	6.58385285	0.308	0.002	0	NK cell	Trdv2-2
0	7.22352096	0.297	0.001	0	NK cell	Gm43647
0	5.58244663	0.765	0.016	0	Prolif/HSPC	Knl1
0	5.51303033	0.728	0.016	0	Prolif/HSPC	Spc24
0	5.60706707	0.694	0.015	0	Prolif/HSPC	Kif15
0	5.86789338	0.564	0.009	0	Prolif/HSPC	Melk
0	5.5893924	0.558	0.009	0	Prolif/HSPC	Aspm
0	5.51797576	0.555	0.01	0	Prolif/HSPC	Bub1
0	5.70431348	0.531	0.008	0	Prolif/HSPC	Kif2c
0	6.48862762	0.529	0.006	0	Prolif/HSPC	Mtfr2
0	5.79480199	0.523	0.007	0	Prolif/HSPC	Pimreg
0	5.50613684	0.46	0.007	0	Prolif/HSPC	Kif14
0	7.30668474	0.452	0.005	0	Prolif/HSPC	Prtn3
0	5.54198908	0.434	0.007	0	Prolif/HSPC	Mcm10
0	5.81215248	0.408	0.007	0	Prolif/HSPC	Iqgap3
0	5.99136605	0.39	0.005	0	Prolif/HSPC	BC030867
0	5.74334483	0.377	0.008	0	Prolif/HSPC	Gria3
0	5.59432933	0.374	0.006	0	Prolif/HSPC	Troap
0	5.9135471	0.342	0.006	0	Prolif/HSPC	Bex6
0	9.07414978	0.335	0.002	0	Prolif/HSPC	Ctsg
0	6.73325424	0.279	0.003	0	Prolif/HSPC	Elane
0	8.5805065	0.26	0.001	0	Prolif/HSPC	Mpo
0	5.81392989	0.948	0.04	0	Monocyte	Gm36161
0	4.84267342	0.985	0.094	0	Monocyte	Lgals3
0	5.30081052	0.916	0.042	0	Monocyte	Gm21188
0	4.84245606	0.927	0.053	0	Monocyte	Emilin2

bioRxiv preprint doi: https://doi.org/10.64898/2025.12.08.693074; this version posted December 11, 2025. The copyright holder for this preprint (which was not certified by peer review) is the author/funder, who has granted bioRxiv a license to display the preprint in perpetuity. It is made available under aCC-BY 4.0 International license.

0	5.22208495	0.933	0.067	0	Monocyte	Plac8
0	5.20107673	0.781	0.034	0	Monocyte	Gpr141
0	5.93802258	0.756	0.026	0	Monocyte	AI839979
0	6.5267081	0.698	0.014	0	Monocyte	Gm9733
0	5.1709648	0.697	0.03	0	Monocyte	Krt80
0	4.82095263	0.691	0.027	0	Monocyte	Gm15987
0	7.63909647	0.627	0.005	0	Monocyte	Arhgef37
0	7.03246257	0.619	0.012	0	Monocyte	Apoc2
0	6.10413655	0.614	0.013	0	Monocyte	Sirpb1c
0	6.8339453	0.547	0.007	0	Monocyte	F10
0	5.95539216	0.546	0.011	0	Monocyte	Galnt9
0	7.36386209	0.466	0.004	0	Monocyte	Ms4a8a
0	7.01026248	0.37	0.009	0	Monocyte	Adgre4
0	5.97871502	0.326	0.008	0	Monocyte	Serpinb2
0	6.41118043	0.318	0.005	0	Monocyte	Vcan
0	4.8859993	0.323	0.012	0	Monocyte	Mefv
0	9.17364385	0.812	0.013	0	FLC	Ccl19
0	5.97337937	0.796	0.036	0	FLC	Col3a1
0	5.07487748	0.698	0.046	0	FLC	Prrx1
0	5.76877307	0.636	0.027	0	FLC	Angpt2
0	6.64292426	0.568	0.022	0	FLC	Steap4
0	5.43055585	0.516	0.025	0	FLC	Angpt1
0	4.92013683	0.501	0.03	0	FLC	Pawr
0	6.13858405	0.483	0.012	0	FLC	Adra2a
0	4.87808898	0.464	0.02	0	FLC	Aoc3
0	5.14413629	0.408	0.015	0	FLC	Ccdc3
0	9.65691823	0.347	0.001	0	FLC	Colec11
0	4.79224059	0.312	0.016	0	FLC	Loxl2
0	4.86705918	0.306	0.015	0	FLC	Susd2
0	4.82644766	0.296	0.017	0	FLC	Wdr86
0	5.46876885	0.285	0.008	0	FLC	Kcnt2
0	6.38612044	0.268	0.004	0	FLC	Nnmt
0	8.60850331	0.26	0.001	0	FLC	Agtr1a
0	5.43499096	0.265	0.011	0	FLC	Alx4
0	5.55670756	0.261	0.009	0	FLC	Col5a1
0	5.31836502	0.261	0.014	0	FLC	Matn2
0	12.3715974	0.693	0.002	0	FB	Igfbp6
0	12.9364598	0.637	0	0	FB	Mpzl2
0	16.386895	0.632	0	0	FB	Slc47a1
0	14.2973511	0.595	0	0	FB	Slc26a7
0	14.7164181	0.589	0	0	FB	Tspan11
0	12.0149145	0.516	0	0	FB	Itgbl1
0	11.5093158	0.491	0.001	0	FB	Adamtsl3
0	13.5165921	0.47	0	0	FB	Cpz
0	10.8714719	0.442	0.001	0	FB	Crabp2
0	13.1464817	0.392	0	0	FB	Shisa3
0	13.6894115	0.378	0	0	FB	Sfrp4
0	11.8043696	0.367	0.001	0	FB	Lum
0	11.0848802	0.337	0.001	0	FB	Cemip
0	16.7706677	0.316	0	0	FB	Cyp2f2
0	10.9539701	0.311	0	0	FB	Prdm6
0	12.2928491	0.309	0	0	FB	Fam180a
0	10.9887549	0.306	0	0	FB	Prmt8
0	11.8124989	0.303	0	0	FB	Platr15
0	11.9920639	0.297	0	0	FB	Mfap4
0	11.0242356	0.268	0	0	FB	Prg4
0	15.2281822	0.996	0	0	OL	Mog
0	13.404504	1	0.006	0	OL	Plp1
0	12.9400948	0.994	0.001	0	OL	Mobp
0	12.6139158	0.992	0.001	0	OL	Stmn4
0	13.3221001	0.99	0	0	OL	Ugt8a
0	14.5733879	0.99	0	0	OL	Fa2h
0	14.8015534	0.986	0	0	OL	Ermn
0	12.0390723	0.976	0.001	0	OL	Cntn2
0	13.6414389	0.962	0	0	OL	Myrf
0	13.5964962	0.96	0	0	OL	Gjb1
0	11.5628252	0.958	0.001	0	OL	Nkain1

bioRxiv preprint doi: https://doi.org/10.64898/2025.12.08.693074; this version posted December 11, 2025. The copyright holder for this preprint (which was not certified by peer review) is the author/funder, who has granted bioRxiv a license to display the preprint in perpetuity. It is made available under aCC-BY 4.0 International license.

0	15.3640711	0.95	0	0	OL	Opalin
0	12.2633857	0.938	0.001	0	OL	Kcna1
0	13.236998	0.903	0	0	OL	Hapln2
0	13.1142003	0.879	0	0	OL	C030029H02Rik
0	11.748553	0.685	0	0	OL	Sec14l5
0	11.7809071	0.651	0	0	OL	Gm42756
0	12.6448057	0.506	0	0	OL	A230001M10Rik
0	13.5272613	0.49	0	0	OL	Cyp2j12
0	11.4722918	0.454	0	0	OL	Sgk2
0	6.56573424	0.753	0.021	0	ILC	Gata3
0	7.38634175	0.642	0.004	0	ILC	Tnfsf11
0	8.61037216	0.603	0.003	0	ILC	Tcrg-C1
0	6.50829009	0.605	0.018	0	ILC	Rnf128
0	5.97401979	0.584	0.018	0	ILC	Il1rl1
0	6.32820472	0.547	0.008	0	ILC	Podnl1
0	7.86460674	0.469	0.009	0	ILC	Trdv4
0	8.80429821	0.341	0.001	0	ILC	Arg1
0	6.77700867	0.334	0.002	0	ILC	Il9r
0	5.76004947	0.321	0.006	0	ILC	Dach2
0	7.53906031	0.31	0.004	0	ILC	Actn2
0	6.77796163	0.308	0.004	0	ILC	Stc2
0	6.1041188	0.308	0.005	0	ILC	Ccr8
0	8.46324596	0.299	0.001	0	ILC	Nmur1
0	6.81331971	0.302	0.004	0	ILC	Rln3
0	7.71999729	0.299	0.004	0	ILC	Rorc
0	6.91909098	0.299	0.005	0	ILC	Aqp3
0	6.70229756	0.293	0.004	0	ILC	Il17rb
0	6.32997507	0.278	0.007	0	ILC	Ly6g5b
0	8.77647204	0.254	0	0	ILC	Cd163l1
0	12.1235657	0.995	0.001	0	cpEpithelial	Folr1
0	14.3099714	1	0.007	0	cpEpithelial	Ttr
0	14.3313065	0.985	0	0	cpEpithelial	Kcne2
0	13.4297498	0.98	0	0	cpEpithelial	Sostdc1
0	14.5378348	0.971	0	0	cpEpithelial	2900040C04Rik
0	12.5050907	0.956	0	0	cpEpithelial	Atp2b3
0	13.07419	0.954	0	0	cpEpithelial	Htr2c
0	11.6802438	0.937	0.001	0	cpEpithelial	Car12
0	11.9715502	0.922	0	0	cpEpithelial	Slc4a5
0	11.3723443	0.92	0.001	0	cpEpithelial	Steap1
0	13.2458354	0.898	0	0	cpEpithelial	Prr32
0	11.2521342	0.854	0	0	cpEpithelial	Vat1l
0	11.954398	0.795	0	0	cpEpithelial	Slc16a8
0	13.8547205	0.793	0	0	cpEpithelial	Defb11
0	11.6651137	0.646	0	0	cpEpithelial	Frmpd1os
0	11.2215157	0.6	0	0	cpEpithelial	Oca2
0	11.6078234	0.354	0	0	cpEpithelial	Lmx1a
0	11.3155975	0.298	0	0	cpEpithelial	Gm14051
0	13.6278106	0.266	0	0	cpEpithelial	Sult1c2
0	13.9346151	0.261	0	0	cpEpithelial	4933429O19Rik
0	12.932226	1	0.001	0	Astrocyte	Bcan
0	13.2701986	1	0.001	0	Astrocyte	Ntsr2
0	12.5832702	1	0.002	0	Astrocyte	Gpr37l1
0	11.0263444	1	0.019	0	Astrocyte	Aldoc
0	11.3171792	0.979	0.001	0	Astrocyte	Gm3764
0	13.6340236	0.977	0.001	0	Astrocyte	Slc6a11
0	11.143983	0.964	0.002	0	Astrocyte	Aqp4
0	11.6438311	0.964	0.003	0	Astrocyte	Btbd17
0	12.1841305	0.847	0.001	0	Astrocyte	Dbx2
0	11.2894992	0.806	0.003	0	Astrocyte	Itih3
0	12.3763144	0.785	0	0	Astrocyte	Gm6145
0	11.1269989	0.767	0.001	0	Astrocyte	AW047730
0	12.0151382	0.744	0.002	0	Astrocyte	Agt
0	12.1793477	0.725	0	0	Astrocyte	Gabrg1
0	11.5199574	0.619	0.001	0	Astrocyte	Grin2c
0	12.0715015	0.614	0	0	Astrocyte	Etnppl
0	11.4572446	0.562	0.001	0	Astrocyte	Cyp4f15
0	11.2105315	0.544	0	0	Astrocyte	Gdpd2

bioRxiv preprint doi: https://doi.org/10.64898/2025.12.08.693074; this version posted December 11, 2025. The copyright holder for this preprint (which was not certified by peer review) is the author/funder, who has granted bioRxiv a license to display the preprint in perpetuity. It is made available under aCC-BY 4.0 International license.

0	11.4289798	0.443	0	0	Astrocyte	Wnt7a
0	11.6107008	0.288	0	0	Astrocyte	Fam181a
0	8.90711022	0.95	0.011	0	pDC	Cox6a2
0	7.55892843	0.936	0.012	0	pDC	Ccr9
0	7.39087687	0.927	0.017	0	pDC	Upb1
0	7.1637102	0.922	0.016	0	pDC	Pacsin1
0	9.73860624	0.9	0.003	0	pDC	Cd300c
0	7.39461509	0.849	0.013	0	pDC	Eldr
0	7.38002481	0.808	0.017	0	pDC	Gm34680
0	10.6101781	0.763	0.001	0	pDC	Atp2a1
0	12.4488287	0.731	0	0	pDC	Gm21762
0	12.2912443	0.717	0	0	pDC	Klk1
0	7.66745413	0.571	0.007	0	pDC	Cd209d
0	9.15561114	0.548	0.002	0	pDC	Grm8
0	9.96854011	0.525	0.001	0	pDC	Havcr1
0	7.24698736	0.525	0.006	0	pDC	Prss30
0	9.53055638	0.466	0.001	0	pDC	Foxr1
0	7.53948166	0.411	0.003	0	pDC	Hoxa7
0	9.23151554	0.393	0.001	0	pDC	Klk1b27
0	8.45899951	0.306	0.001	0	pDC	Fbxl13
0	9.6730006	0.279	0.001	0	pDC	Gm12253
0	7.03124102	0.279	0.002	0	pDC	Robo3
0	14.8044388	0.856	0	0	Erythroid	Slc4a1
0	12.1413222	0.856	0.001	0	Erythroid	Spta1
0	13.7217247	0.856	0.001	0	Erythroid	Rhd
0	14.5557555	0.849	0	0	Erythroid	Gypa
0	12.6641973	0.849	0.001	0	Erythroid	Kel
0	12.9456148	0.842	0	0	Erythroid	Hbb-bs
0	13.1191328	0.842	0	0	Erythroid	Hba-a1
0	12.5496116	0.835	0	0	Erythroid	Hba-a2
0	14.0552772	0.827	0	0	Erythroid	Hbb-bt
0	13.2664601	0.827	0	0	Erythroid	Alas2
0	13.3184444	0.827	0.002	0	Erythroid	Hemgn
0	13.1435766	0.82	0	0	Erythroid	Cldn13
0	13.5172709	0.813	0	0	Erythroid	Epb42
0	12.0275484	0.813	0.001	0	Erythroid	Rhag
0	12.243625	0.799	0	0	Erythroid	Trim10
0	11.6751185	0.799	0.001	0	Erythroid	Tspo2
0	13.7027959	0.777	0	0	Erythroid	Hbq1b
0	11.9177375	0.712	0	0	Erythroid	Btnl10
0	14.766977	0.317	0	0	Erythroid	Apol11b
0	11.941497	0.259	0	0	Erythroid	Gm20161
0	14.5213441	0.436	0	0	Neural	Tmem212
0	14.9445639	0.436	0	0	Neural	Ccdc153
0	13.6350111	0.436	0	0	Neural	Fam183b
0	11.8432832	0.427	0	0	Neural	Iqca
0	11.9239966	0.427	0	0	Neural	Fam216b
0	11.8132247	0.418	0.001	0	Neural	Hydin
0	11.8191172	0.391	0	0	Neural	Lrrc74b
0	12.4296072	0.382	0	0	Neural	Gm10714
0	13.1157837	0.373	0	0	Neural	Acot5
0	12.6347816	0.373	0	0	Neural	1700024G13Rik
0	14.1943158	0.355	0	0	Neural	Npy
0	12.3412567	0.327	0	0	Neural	Stoml3
0	12.4985546	0.327	0	0	Neural	Lrrc43
0	13.8733036	0.327	0	0	Neural	Got1l1
0	14.2104445	0.327	0	0	Neural	Zfp474
0	11.842276	0.318	0	0	Neural	Dthd1
0	11.5233047	0.309	0	0	Neural	Ccdc121
0	13.7495327	0.291	0	0	Neural	3300002A11Rik
0	11.6831206	0.282	0	0	Neural	Dlx6os1
0	11.8953337	0.273	0	0	Neural	Akap14
0	11.154033	0.96	0.001	0	MC/BP	Cyp11a1
0	10.9993265	0.96	0.001	0	MC/BP	Cd200r3
0	10.4218104	0.94	0.001	0	MC/BP	Ms4a2
0	8.16327928	0.92	0.002	0	MC/BP	Cpa3
0	10.7248465	0.86	0.001	0	MC/BP	Fcer1a

bioRxiv preprint doi: https://doi.org/10.64898/2025.12.08.693074; this version posted December 11, 2025. The copyright holder for this preprint (which was not certified by peer review) is the author/funder, who has granted bioRxiv a license to display the preprint in perpetuity. It is made available under aCC-BY 4.0 International license.

0	11.9408925	0.84	0.001	0	MC/BP	Csrp3
0	11.1284122	0.78	0.001	0	MC/BP	Alox15
0	8.67715336	0.72	0.001	0	MC/BP	Mcpt8
0	10.0741598	0.72	0.001	0	MC/BP	Hrh4
0	11.1345528	0.68	0	0	MC/BP	Grm6
0	12.6912127	0.58	0	0	MC/BP	Gm35147
0	8.57916057	0.56	0	0	MC/BP	Prss34
0	8.86987848	0.56	0.002	0	MC/BP	D430036J16Rik
0	10.6407331	0.48	0.001	0	MC/BP	1110028F11Rik
0	10.1645317	0.46	0.001	0	MC/BP	Alox8
0	9.04967917	0.44	0	0	MC/BP	Slc6a4
0	9.70705619	0.32	0	0	MC/BP	Rnf39
0	10.056887	0.28	0	0	MC/BP	Oit3
0	13.0608401	0.28	0	0	MC/BP	1700041M19Rik
0	8.24364162	0.28	0.001	0	MC/BP	Angptl8

Table S1. Cell type markers for scRNAseq atlas.
Top 20 genes defining each broad cell type in the scRNA-seq atlas (**Figure 1B**). Fine annotation and resulting markers are located in **Figure S1**.

bioRxiv preprint doi: https://doi.org/10.64898/2025.12.08.693074; this version posted December 11, 2025. The copyright holder for this preprint (which was not certified by peer review) is the author/funder, who has granted bioRxiv a license to display the preprint in perpetuity. It is made available under aCC-BY 4.0 International license.

Table S2:

gene	p_val	avg_log2FC	pct.1	pct.2	p_val_adj
Dbp	1.69E-173	-2.3720621	1	1	3.90E-169
Fkbp5	2.00E-78	-2.3640932	1	1	4.61E-74
Nr1d2	6.40E-53	-1.2047761	1	1	1.48E-48
Sult1a1	5.16E-42	-0.8560032	1	1	1.19E-37
Rbm3	2.20E-39	-0.9535314	1	1	5.08E-35
Pim3	9.56E-31	-0.8913226	1	1	2.21E-26
Ucp2	4.64E-29	-0.7296599	1	1	1.07E-24
Ccl12	1.05E-26	-0.6910647	1	1	2.42E-22
Tspan4	2.87E-24	-0.707641	1	1	6.63E-20
Glul	7.10E-22	-0.6517405	1	1	1.64E-17
Tef	2.40E-21	-0.7477913	1	1	5.55E-17
Ogfrl1	3.31E-21	-0.6223906	1	1	7.65E-17
Fcgr1	9.54E-21	0.51311984	1	1	2.21E-16
Actg1	1.38E-20	-0.4762861	1	1	3.20E-16
Klf4	3.44E-19	-1.2765923	1	1	7.95E-15
Abca1	1.05E-18	0.56309022	1	1	2.43E-14
Zeb2	6.46E-17	0.43759089	1	1	1.49E-12
Ms4a4a	6.80E-16	-0.857031	1	1	1.57E-11
Calr	3.06E-15	0.55811136	1	1	7.08E-11
Clec2d	3.55E-15	0.54560312	1	1	8.21E-11
Per3	4.68E-15	-0.6191829	1	1	1.08E-10
Nr1d1	2.77E-14	-1.4790172	1	1	6.41E-10
Paox	5.74E-14	-0.4972551	1	1	1.33E-09
Thra	5.77E-14	-0.5604012	1	1	1.33E-09
Lyve1	9.81E-14	-0.5222946	1	1	2.27E-09
Arntl	4.09E-13	0.54200754	1	1	9.46E-09
Tlr13	6.26E-13	-0.5302653	1	1	1.45E-08
Fyb	1.56E-12	0.40145201	1	1	3.62E-08
Marcks	1.72E-12	0.38168882	1	1	3.97E-08
Pim1	2.82E-12	0.54836739	1	1	6.51E-08
Lifr	3.42E-12	-0.4583516	1	1	7.90E-08
Bri3	3.95E-12	-0.3595776	1	1	9.12E-08
Ptpre	4.82E-12	0.50739004	1	1	1.11E-07
Cirbp	6.45E-12	-0.5535028	1	1	1.49E-07
Thbd	6.56E-12	0.53244159	1	1	1.52E-07
Ifi203	1.12E-11	0.45380661	1	1	2.59E-07
Ms4a6b	1.61E-11	-0.4405878	1	1	3.71E-07
Tmem37	1.68E-11	-0.4068566	1	1	3.87E-07
Pacsin2	1.37E-10	-0.4195486	1	1	3.16E-06
Ninj1	1.46E-10	-0.3389956	1	1	3.38E-06
Ftl1	1.50E-10	-0.2412377	1	1	3.47E-06
Ltc4s	2.49E-10	-0.2761432	1	1	5.77E-06
Rab7b	2.87E-10	-0.5227186	1	1	6.63E-06
Chka	3.08E-10	-0.4818887	1	1	7.12E-06
Sla	3.23E-10	-0.4843512	1	1	7.46E-06
Dhx9	3.54E-10	-0.487822	1	1	8.19E-06
Selenop	4.14E-10	-0.3390234	1	1	9.58E-06
B4galt1	6.97E-10	-0.4721215	1	1	1.61E-05
Dynll1	9.20E-10	0.53038966	1	1	2.13E-05
Cd68	9.54E-10	-0.2775491	1	1	2.21E-05
Srm	1.02E-09	-0.5231574	1	1	2.36E-05
Ppp1r14b	1.14E-09	-0.4097375	1	1	2.64E-05
Susd6	1.15E-09	0.51305867	1	1	2.67E-05
Eif4a1	1.19E-09	-0.3380774	1	1	2.74E-05
Klf9	1.25E-09	-0.3949288	1	1	2.90E-05
Adgre1	1.49E-09	0.33581534	1	1	3.45E-05
Adrb1	1.53E-09	-0.4593049	1	1	3.53E-05
Dcxr	1.67E-09	-0.4449671	1	1	3.86E-05
Kcnq1ot1	1.82E-09	0.50266058	1	1	4.22E-05
Lst1	1.89E-09	0.42397012	1	1	4.37E-05
Runx1	2.10E-09	0.43296314	1	1	4.85E-05
Meg3	2.44E-09	0.43266503	1	1	5.64E-05
Adam8	2.52E-09	-0.4617471	1	1	5.82E-05
Ptprc	3.25E-09	0.35649864	1	1	7.52E-05

bioRxiv preprint doi: https://doi.org/10.64898/2025.12.08.693074; this version posted December 11, 2025. The copyright holder for this preprint (which was not certified by peer review) is the author/funder, who has granted bioRxiv a license to display the preprint in perpetuity. It is made available under aCC-BY 4.0 International license.

Tbc1d10a	4.17E-09	-0.4526978	1	1	9.65E-05
Tmcc3	7.25E-09	0.47962825	1	1	0.00016775
Slc15a3	7.27E-09	0.46322429	1	1	0.0001681
Itsn1	7.85E-09	0.3656476	1	1	0.00018158
Per1	8.96E-09	-1.5072466	1	1	0.0002071
mt-Cytb	1.26E-08	-0.2603041	1	1	0.00029045
Udp2	1.27E-08	-1.0481953	1	1	0.00029307
Gdap2	1.29E-08	-0.4420762	1	1	0.00029749
Lbx1	1.33E-08	-0.4088787	1	1	0.00030699
Cx3cr1	1.43E-08	1.08744273	1	1	0.00033055
Egfr	1.47E-08	-0.4524619	1	1	0.00034067
Tmod1	1.61E-08	-0.4377463	1	1	0.00037172
Itga4	1.63E-08	0.38707001	1	1	0.00037764
Rcsd1	2.47E-08	-0.3609045	1	1	0.00057102
Hmgcl	2.58E-08	-0.409849	1	1	0.00059622
Plekho2	2.96E-08	0.42714704	1	1	0.00068453
Pmp22	3.08E-08	-0.2881877	1	1	0.00071149
Hif3a	3.53E-08	-0.2917903	1	1	0.00081636
Slc25a10	4.69E-08	-0.4200243	1	1	0.00108532
Rhob	8.52E-08	-0.8539209	1	1	0.00196954
Trps1	8.94E-08	0.42385158	1	1	0.00206645
Pnpla2	8.99E-08	-0.4005112	1	1	0.00207938
Frmd4b	9.89E-08	-0.3041318	1	1	0.00228759
Gm29291	9.98E-08	-0.4294377	1	1	0.00230859
Parp14	1.23E-07	0.41104479	1	1	0.00284364
Bcl3	1.33E-07	0.43767441	1	1	0.00307199
Ccnd3	1.35E-07	-0.3897464	1	1	0.00312029
Cyba	1.60E-07	-0.2373442	1	1	0.0037109
Zeb2os	1.71E-07	-0.3882553	1	1	0.00396276
Kcnk6	1.81E-07	-0.3852223	1	1	0.00419118
Slfn5	1.84E-07	0.39551431	1	1	0.0042567
Vat1	1.95E-07	-0.3972826	1	1	0.00451437
Abca9	2.07E-07	0.27421129	1	1	0.00478312
Hspa8	2.24E-07	0.31260091	1	1	0.00518728
Zbtb16	2.28E-07	-0.3257315	1	1	0.00526935
Ifi213	2.49E-07	0.41549121	1	1	0.00576062
Cmklr1	2.91E-07	-0.3437484	1	1	0.00672823
Slc25a5	2.98E-07	-0.3030526	1	1	0.00688868
Slco2b1	2.99E-07	0.29341293	1	1	0.00690408
Atp6v0a1	3.05E-07	0.3246665	1	1	0.00705314
Ednrb	3.22E-07	-0.3268769	1	1	0.00743474
Afap1l1	3.31E-07	-0.3519194	1	1	0.00764851
Trpv4	3.76E-07	-0.3732284	1	1	0.00869976
Prdx1	3.84E-07	-0.2567964	1	1	0.00888987
Sgk1	3.91E-07	-0.4010854	1	1	0.00904804
Dab2	4.20E-07	0.21136511	1	1	0.00970501
Malat1	4.64E-07	0.29069634	1	1	0.01072049
45907	5.36E-07	-0.3176111	1	1	0.01238711
Actb	5.45E-07	-0.2352446	1	1	0.01259515
Mef2c	5.74E-07	0.27036992	1	1	0.01326341
Rapsn	5.82E-07	-0.3951172	1	1	0.01345703
Hmox1	6.51E-07	-0.3313336	1	1	0.01505593
Rac3	6.52E-07	-0.4104609	1	1	0.01507278
Ctsa	6.61E-07	-0.2496255	1	1	0.01529004
5033421B08Rik	6.94E-07	-0.3759339	1	1	0.01604098
Gm15635	8.45E-07	0.3670391	1	1	0.01953086
Hspa5	9.13E-07	0.26311088	1	1	0.02110147
S1pr1	9.21E-07	0.35652759	1	1	0.02129558
Mycbp2	9.84E-07	0.32209651	1	1	0.02276402
Myc	9.88E-07	-0.3754003	1	1	0.0228407
Pfn1	1.02E-06	-0.2219362	1	1	0.0235143
Pou2f2	1.07E-06	0.38597016	1	1	0.02466247
Sh3bgrl3	1.11E-06	-0.3079928	1	1	0.02573791
CAAA01147332.1	1.15E-06	-0.28766	1	1	0.02670166
Ms4a6c	1.17E-06	-0.5436771	1	1	0.02710722
Notch2	1.18E-06	0.36194523	1	1	0.02725608
Dock8	1.30E-06	0.32227372	1	1	0.03011545

bioRxiv preprint doi: https://doi.org/10.64898/2025.12.08.693074; this version posted December 11, 2025. The copyright holder for this preprint (which was not certified by peer review) is the author/funder, who has granted bioRxiv a license to display the preprint in perpetuity. It is made available under aCC-BY 4.0 International license.

Lrrc25	1.34E-06	0.28231511	1	1	0.0309321
Ncf1	1.41E-06	-0.3495676	1	1	0.03269517
Chd3	1.42E-06	0.40268129	1	1	0.0327831
Fbl	1.43E-06	-0.3577826	1	1	0.03303516
Cflar	1.44E-06	0.33523526	1	1	0.03328337
Prex1	1.56E-06	0.34177959	1	1	0.03617607
Sap30	1.70E-06	-0.3874487	1	1	0.03928514
Aftph	1.72E-06	0.32160857	1	1	0.03985409
Ms4a6d	1.80E-06	-0.2825788	1	1	0.04165377
Gdpd1	1.86E-06	-0.3569335	1	1	0.04307223
Zfp217	1.89E-06	0.35334489	1	1	0.04362361
Kmt2d	1.97E-06	0.38163954	1	1	0.04560042
Pla2g7	2.00E-06	-0.3324563	1	1	0.04631375

Table S2. Pseudobulk comparison of BAMs from ZT0 and ZT12.
Results of pseudobulk analysis comparing BAMs at ZT0 and ZT12, filtered for adjusted p-value <0.05. Negative values represent genes upregulated at ZT12 relative to ZT0.

bioRxiv preprint doi: https://doi.org/10.64898/2025.12.08.693074; this version posted December 11, 2025. The copyright holder for this preprint (which was not certified by peer review) is the author/funder, who has granted bioRxiv a license to display the preprint in perpetuity. It is made available under aCC-BY 4.0 International license.

Table S3:

Gene set	Ingenuity Canonical Pathways	$-log_{10}pval$
Upregulated ZT0	Immunogenic Cell Death Signaling Pathway	8.91E+00
Upregulated ZT0	HSP90 chaperone cycle for steroid hormone receptors in the presence of ligand	8.72E+00
Upregulated ZT0	Stress Granule Signaling Pathway	7.89E+00
Upregulated ZT0	Unfolded protein response	6.31E+00
Upregulated ZT0	Cellular response to heat stress	5.99E+00
Upregulated ZT0	RHO GTPase cycle	5.82E+00
Upregulated ZT0	ESR-mediated signaling	5.81E+00
Upregulated ZT0	NOD1/2 Signaling Pathway	5.78E+00
Upregulated ZT0	Cyclophilin Signaling Pathway	5.76E+00
Upregulated ZT0	ATF6 (ATF6-alpha) activates chaperone genes	5.59E+00
Upregulated ZT0	Semaphorin interactions	5.25E+00
Upregulated ZT0	Role of PKR in Interferon Induction and Antiviral Response	5.22E+00
Upregulated ZT0	Protein Ubiquitination Pathway	5.19E+00
Upregulated ZT0	Natural Killer Cell Signaling	4.89E+00
Upregulated ZT0	Aldosterone Signaling in Epithelial Cells	4.82E+00
Upregulated ZT0	Neutrophil degranulation	4.33E+00
Upregulated ZT0	UFMylation Signaling Pathway	4.27E+00
Upregulated ZT0	Endoplasmic Reticulum Stress Pathway	4.26E+00
Upregulated ZT0	Heme signaling	3.93E+00
Upregulated ZT0	Xenobiotic Metabolism AHR Signaling Pathway	3.68E+00
Upregulated ZT0	Nonsense-Mediated Decay (NMD)	3.68E+00
Upregulated ZT0	Multiple Sclerosis Signaling Pathway	3.64E+00
Upregulated ZT0	Circadian Clock	3.44E+00
Upregulated ZT0	IL-27 Signaling Pathway	3.43E+00
Upregulated ZT0	ISGylation Signaling Pathway	3.40E+00
Upregulated ZT0	Cachexia Signaling Pathway	3.34E+00
Upregulated ZT0	Pre-NOTCH Expression and Processing	3.30E+00
Upregulated ZT0	Cerebral Malformation Signaling Pathway	3.28E+00
Upregulated ZT0	Binding and Uptake of Ligands by Scavenger Receptors	3.23E+00
Upregulated ZT0	Response of EIF2AK4 (GCN2) to amino acid deficiency	3.15E+00
Upregulated ZT0	Aryl Hydrocarbon Receptor Signaling	2.95E+00
Upregulated ZT0	Neuroinflammation Signaling Pathway	2.95E+00
Upregulated ZT0	Retinoic acid Mediated Apoptosis Signaling	2.90E+00
Upregulated ZT0	Hypoxia Signaling in the Cardiovascular System	2.87E+00
Upregulated ZT0	EIF2 Signaling	2.86E+00
Upregulated ZT0	Chaperone Mediated Autophagy	2.85E+00
Upregulated ZT0	Glucocorticoid Receptor Signaling	2.80E+00
Upregulated ZT0	Neuregulin Signaling	2.78E+00
Upregulated ZT0	Coronavirus Replication Pathway	2.71E+00
Upregulated ZT0	Class I MHC mediated antigen processing and presentation	2.68E+00
Upregulated ZT0	Aryl hydrocarbon receptor signalling	2.63E+00
Upregulated ZT0	Coronavirus Pathogenesis Pathway	2.62E+00
Upregulated ZT0	Fcgamma receptor (FCGR) dependent phagocytosis	2.61E+00
Upregulated ZT0	Eukaryotic Translation Termination	2.56E+00
Upregulated ZT0	Eukaryotic Translation Elongation	2.55E+00
Upregulated ZT0	Actin Nucleation by ARP-WASP Complex	2.54E+00
Upregulated ZT0	EPH-Ephrin signaling	2.52E+00
Upregulated ZT0	Histone Modification Signaling Pathway	2.52E+00
Upregulated ZT0	NRF2-mediated Oxidative Stress Response	2.50E+00
Upregulated ZT0	Death Receptor Signaling	2.48E+00
Upregulated ZT0	Semaphorin Signaling in Neurons	2.46E+00
Upregulated ZT0	Transcriptional regulation by RUNX3	2.44E+00
Upregulated ZT0	Myogenesis	2.44E+00
Upregulated ZT0	Zygotic genome activation (ZGA)	2.40E+00
Upregulated ZT0	RIPK1-mediated regulated necrosis	2.40E+00
Upregulated ZT0	Role of Pattern Recognition Receptors in Recognition of Bacteria and Viruses	2.36E+00
Upregulated ZT0	PPARŒ±/RXRŒ± Activation	2.35E+00
Upregulated ZT0	mRNA 3 Prime End Processing Signaling Pathway	2.34E+00
Upregulated ZT0	Mitotic Prometaphase	2.30E+00
Upregulated ZT0	Interleukin-4 and Interleukin-13 signaling	2.29E+00
Upregulated ZT0	SRP-dependent cotranslational protein targeting to membrane	2.26E+00
Upregulated ZT0	Activin Inhibin Signaling Pathway	2.26E+00
Upregulated ZT0	PI3K/AKT Signaling	2.26E+00

bioRxiv preprint doi: https://doi.org/10.64898/2025.12.08.693074; this version posted December 11, 2025. The copyright holder for this preprint (which was not certified by peer review) is the author/funder, who has granted bioRxiv a license to display the preprint in perpetuity. It is made available under aCC-BY 4.0 International license.

Upregulated ZT0	PPAR Signaling	2.26E+00
Upregulated ZT0	eNOS Signaling	2.24E+00
Upregulated ZT0	Regulation of Actin-based Motility by Rho	2.22E+00
Upregulated ZT0	Signaling by ROBO receptors	2.22E+00
Upregulated ZT0	Ephrin Receptor Signaling	2.20E+00
Upregulated ZT0	Selenoamino acid metabolism	2.19E+00
Upregulated ZT0	TREM1 Signaling	2.19E+00
Upregulated ZT0	Regulation of eIF4 and p70S6K Signaling	2.18E+00
Upregulated ZT0	Role of Macrophages, Fibroblasts and Endothelial Cells in Rheumatoid Arthritis	2.16E+00
Upregulated ZT0	Ribosomal Quality Control Signaling Pathway	2.15E+00
Upregulated ZT0	Trafficking and processing of endosomal TLR	2.15E+00
Upregulated ZT0	Eukaryotic Translation Initiation	2.15E+00
Upregulated ZT0	Chaperone Mediated Autophagy Signaling Pathway	2.12E+00
Upregulated ZT0	Caveolar-mediated Endocytosis Signaling	2.08E+00
Upregulated ZT0	RHOGDI Signaling	2.05E+00
Upregulated ZT0	Role of JAK family kinases in IL-6-type Cytokine Signaling	2.04E+00
Upregulated ZT0	Chromatin organization	2.04E+00
Upregulated ZT0	Immunoregulatory interactions between a Lymphoid and a non-Lymphoid cell	2.02E+00
Upregulated ZT0	Metabolism of nitric oxide: NOS3 activation and regulation	2.02E+00
Upregulated ZT0	Phagosome Formation	1.98E+00
Upregulated ZT0	Pyroptosis Signaling Pathway	1.96E+00
Upregulated ZT0	Caspase activation via Death Receptors in the presence of ligand	1.96E+00
Upregulated ZT0	GAIT Translation Signaling Pathway	1.95E+00
Upregulated ZT0	BAG2 Signaling Pathway	1.94E+00
Upregulated ZT0	PEDF Signaling	1.89E+00
Upregulated ZT0	TCF dependent signaling in response to WNT	1.88E+00
Upregulated ZT0	MicroRNA Biogenesis Signaling Pathway	1.87E+00
Upregulated ZT0	ABRA Signaling Pathway	1.84E+00
Upregulated ZT0	Actin Cytoskeleton Signaling	1.83E+00
Upregulated ZT0	NR1H2 and NR1H3-mediated signaling	1.83E+00
Upregulated ZT0	Mitotic G2-G2/M phases	1.80E+00
Upregulated ZT0	Cell Cycle: G2/M DNA Damage Checkpoint Regulation	1.78E+00
Upregulated ZT0	Leukocyte Extravasation Signaling	1.78E+00
Upregulated ZT0	Acute Myeloid Leukemia Signaling	1.77E+00
Upregulated ZT0	Unfolded Protein Response (UPR)	1.76E+00
Upregulated ZT0	RHO GTPases Activate ROCKs	1.76E+00
Upregulated ZT0	Transcriptional regulation by RUNX2	1.76E+00
Upregulated ZT0	Irritable Bowel Syndrome Signaling Pathway	1.73E+00
Upregulated ZT0	Inflammasome pathway	1.72E+00
Upregulated ZT0	Semaphorin Neuronal Repulsive Signaling Pathway	1.71E+00
Upregulated ZT0	Clathrin-mediated Endocytosis Signaling	1.66E+00
Upregulated ZT0	Formation of paraxial mesoderm	1.64E+00
Upregulated ZT0	Factors Promoting Cardiogenesis in Vertebrates	1.63E+00
Upregulated ZT0	PTEN Signaling	1.63E+00
Upregulated ZT0	Xenobiotic Metabolism Signaling	1.60E+00
Upregulated ZT0	Protein Kinase A Signaling	1.60E+00
Upregulated ZT0	Nephrin family interactions	1.60E+00
Upregulated ZT0	Epithelial Adherens Junction Signaling	1.58E+00
Upregulated ZT0	Calcium Signaling	1.57E+00
Upregulated ZT0	Regulation of RUNX1 Expression and Activity	1.57E+00
Upregulated ZT0	Xenobiotic Metabolism PXR Signaling Pathway	1.56E+00
Upregulated ZT0	Signaling by VEGF	1.56E+00
Upregulated ZT0	Telomerase Signaling	1.56E+00
Upregulated ZT0	HOTAIR Regulatory Pathway	1.55E+00
Upregulated ZT0	Chronic Myeloid Leukemia Signaling	1.54E+00
Upregulated ZT0	Estrogen Receptor Signaling	1.52E+00
Upregulated ZT0	Major pathway of rRNA processing in the nucleolus and cytosol	1.51E+00
Upregulated ZT0	Antigen Presentation Pathway	1.50E+00
Upregulated ZT0	Role of Hypercytokinemia/hyperchemokinemia in the Pathogenesis of Influenza	1.50E+00
Upregulated ZT0	Macrophage Alternative Activation Signaling Pathway	1.50E+00
Upregulated ZT0	Processing of Capped Intron-Containing Pre-mRNA	1.48E+00
Upregulated ZT0	Prostate Cancer Signaling	1.47E+00
Upregulated ZT0	DDX58/IFIH1-mediated induction of interferon-alpha/beta	1.47E+00
Upregulated ZT0	Mitotic Roles of Polo-Like Kinase	1.47E+00
Upregulated ZT0	Xenobiotic Metabolism CAR Signaling Pathway	1.45E+00
Upregulated ZT0	TP53 Regulates Transcription of DNA Repair Genes	1.45E+00
Upregulated ZT0	Regulation of lipid metabolism by PPARalpha	1.45E+00

bioRxiv preprint doi: https://doi.org/10.64898/2025.12.08.693074; this version posted December 11, 2025. The copyright holder for this preprint (which was not certified by peer review) is the author/funder, who has granted bioRxiv a license to display the preprint in perpetuity. It is made available under aCC-BY 4.0 International license.

Upregulated ZT0	PAK Signaling	1.45E+00
Upregulated ZT0	Osteoarthritis Pathway	1.45E+00
Upregulated ZT0	RHO GTPases activate PKNs	1.44E+00
Upregulated ZT0	Agrin Interactions at Neuromuscular Junction	1.43E+00
Upregulated ZT0	WNT/Œ≤-catenin Signaling	1.43E+00
Upregulated ZT0	Parkinson's Signaling Pathway	1.43E+00
Upregulated ZT0	Cell surface interactions at the vascular wall	1.42E+00
Upregulated ZT0	Toll-like Receptor Cascades	1.41E+00
Upregulated ZT0	Nitric Oxide Signaling in the Cardiovascular System	1.41E+00
Upregulated ZT0	Ion channel transport	1.41E+00
Upregulated ZT0	Nicotinate metabolism	1.39E+00
Upregulated ZT0	DAP12 interactions	1.39E+00
Upregulated ZT0	Axonal Guidance Signaling	1.37E+00
Upregulated ZT0	ERK5 Signaling	1.36E+00
Upregulated ZT0	Ephrin B Signaling	1.36E+00
Upregulated ZT0	VDR/RXR Activation	1.34E+00
Upregulated ZT0	Signaling by NOTCH1	1.31E+00
Upregulated ZT0	MAPK targets/ Nuclear events mediated by MAP kinases	1.31E+00
Upregulated ZT0	D-myo-inositol (1,4,5,6)-Tetrakisphosphate Biosynthesis	1.31E+00
Upregulated ZT0	D-myo-inositol (3,4,5,6)-tetrakisphosphate Biosynthesis	1.31E+00
Upregulated ZT0	Clathrin-mediated endocytosis	1.30E+00
Upregulated ZT12	FcŒ≥ Receptor-mediated Phagocytosis in Macrophages and Monocytes	6.59E+00
Upregulated ZT12	Clathrin-mediated endocytosis	3.53E+00
Upregulated ZT12	Synthesis, secretion, and deacylation of Ghrelin	3.45E+00
Upregulated ZT12	Fcgamma receptor (FCGR) dependent phagocytosis	3.43E+00
Upregulated ZT12	EPH-Ephrin signaling	3.34E+00
Upregulated ZT12	IL-8 Signaling	3.24E+00
Upregulated ZT12	Integrin Signaling	3.21E+00
Upregulated ZT12	Mitochondrial biogenesis	3.05E+00
Upregulated ZT12	Phosphatidylcholine Biosynthesis I	3.00E+00
Upregulated ZT12	Translocation of SLC2A4 (GLUT4) to the plasma membrane	2.97E+00
Upregulated ZT12	Phosphatidylethanolamine Biosynthesis II	2.87E+00
Upregulated ZT12	Protein Sorting Signaling Pathway	2.82E+00
Upregulated ZT12	GPVI-mediated activation cascade	2.72E+00
Upregulated ZT12	TR/RXR Activation	2.70E+00
Upregulated ZT12	Pyroptosis Signaling Pathway	2.61E+00
Upregulated ZT12	Transcriptional regulation of white adipocyte differentiation	2.57E+00
Upregulated ZT12	Circadian Rhythm Signaling	2.57E+00
Upregulated ZT12	TAK1-dependent IKK and NF-kappa-B activation	2.49E+00
Upregulated ZT12	RHO GTPases Activate WASPs and WAVEs	2.47E+00
Upregulated ZT12	TBC/RABGAPs	2.47E+00
Upregulated ZT12	Heme signaling	2.41E+00
Upregulated ZT12	Choline Biosynthesis III	2.37E+00
Upregulated ZT12	Neuroinflammation Signaling Pathway	2.37E+00
Upregulated ZT12	HSP90 chaperone cycle for steroid hormone receptors in the presence of ligand	2.33E+00
Upregulated ZT12	Serotonin Receptor Signaling	2.32E+00
Upregulated ZT12	Macrophage Alternative Activation Signaling Pathway	2.23E+00
Upregulated ZT12	cAMP-mediated signaling	2.20E+00
Upregulated ZT12	GŒ±q Signaling	2.18E+00
Upregulated ZT12	Signaling by VEGF	2.17E+00
Upregulated ZT12	Actin Cytoskeleton Signaling	2.17E+00
Upregulated ZT12	Regulation of Actin-based Motility by Rho	2.16E+00
Upregulated ZT12	Cytoprotection by HMOX1	2.14E+00
Upregulated ZT12	Circadian Clock	2.12E+00
Upregulated ZT12	Signaling by the B Cell Receptor (BCR)	2.09E+00
Upregulated ZT12	Peroxisomal protein import	2.04E+00
Upregulated ZT12	Production of Nitric Oxide and Reactive Oxygen Species in Macrophages	2.01E+00
Upregulated ZT12	Remodeling of Epithelial Adherens Junctions	2.00E+00
Upregulated ZT12	RAF/MAP kinase cascade	1.99E+00
Upregulated ZT12	Leukocyte Extravasation Signaling	1.96E+00
Upregulated ZT12	RAF-independent MAPK1/3 activation	1.95E+00
Upregulated ZT12	Glioma Signaling	1.93E+00
Upregulated ZT12	ILK Signaling	1.93E+00
Upregulated ZT12	Agrin Interactions at Neuromuscular Junction	1.91E+00
Upregulated ZT12	RHO GTPases activate IQGAPs	1.91E+00
Upregulated ZT12	Cachexia Signaling Pathway	1.90E+00
Upregulated ZT12	RHO GTPase cycle	1.88E+00

bioRxiv preprint doi: https://doi.org/10.64898/2025.12.08.693074; this version posted December 11, 2025. The copyright holder for this preprint (which was not certified by peer review) is the author/funder, who has granted bioRxiv a license to display the preprint in perpetuity. It is made available under aCC-BY 4.0 International license.

Upregulated ZT12	IL-10 Signaling	1.86E+00
Upregulated ZT12	fMLP Signaling in Neutrophils	1.86E+00
Upregulated ZT12	BMAL1:CLOCK,NPAS2 activates circadian gene expression	1.84E+00
Upregulated ZT12	Fc epsilon receptor (FCERI) signaling	1.84E+00
Upregulated ZT12	IL-17A Signaling in Fibroblasts	1.83E+00
Upregulated ZT12	VDR/RXR Activation	1.81E+00
Upregulated ZT12	Ephrin A Signaling	1.81E+00
Upregulated ZT12	Phagosome Formation	1.79E+00
Upregulated ZT12	Phase I - Functionalization of compounds	1.72E+00
Upregulated ZT12	ER-Mitochondrial Communication Signaling Pathway	1.72E+00
Upregulated ZT12	Role of Osteoclasts in Rheumatoid Arthritis Signaling Pathway	1.70E+00
Upregulated ZT12	NRF2-mediated Oxidative Stress Response	1.66E+00
Upregulated ZT12	p75 NTR receptor-mediated signalling	1.64E+00
Upregulated ZT12	mTOR Signaling	1.63E+00
Upregulated ZT12	Role of Macrophages, Fibroblasts and Endothelial Cells in Rheumatoid Arthritis	1.63E+00
Upregulated ZT12	Preeclampsia Signaling Pathway	1.63E+00
Upregulated ZT12	Regulation of Cellular Mechanics by Calpain Protease	1.62E+00
Upregulated ZT12	Hepatic Fibrosis Signaling Pathway	1.61E+00
Upregulated ZT12	Transcriptional regulation by the AP-2 (TFAP2) family of transcription factors	1.60E+00
Upregulated ZT12	Actin Nucleation by ARP-WASP Complex	1.59E+00
Upregulated ZT12	Germ Cell-Sertoli Cell Junction Signaling	1.56E+00
Upregulated ZT12	Mitochondrial protein degradation	1.52E+00
Upregulated ZT12	VEGF Signaling	1.52E+00
Upregulated ZT12	Glucocorticoid Receptor Signaling	1.51E+00
Upregulated ZT12	Glioblastoma Multiforme Signaling	1.50E+00
Upregulated ZT12	Irritable Bowel Syndrome Signaling Pathway	1.50E+00
Upregulated ZT12	Sumoylation Pathway	1.48E+00
Upregulated ZT12	Cardiac Hypertrophy Signaling	1.48E+00
Upregulated ZT12	Smooth Muscle Contraction	1.47E+00
Upregulated ZT12	Cargo recognition for clathrin-mediated endocytosis	1.43E+00
Upregulated ZT12	Colorectal Cancer Metastasis Signaling	1.41E+00
Upregulated ZT12	Antioxidant Action of Vitamin C	1.39E+00
Upregulated ZT12	Insulin Secretion Signaling Pathway	1.37E+00
Upregulated ZT12	Sheddase Signaling Pathway	1.37E+00
Upregulated ZT12	Agranulocyte Adhesion and Diapedesis	1.36E+00
Upregulated ZT12	Acetylcholine Receptor Signaling Pathway	1.35E+00
Upregulated ZT12	MYC Mediated Apoptosis Signaling	1.35E+00
Upregulated ZT12	iNOS Signaling	1.35E+00
Upregulated ZT12	Intra-Golgi and retrograde Golgi-to-ER traffic	1.34E+00
Upregulated ZT12	Sleep NREM Signaling Pathway	1.34E+00
Upregulated ZT12	Cholecystokinin/Gastrin-mediated Signaling	1.34E+00
Upregulated ZT12	Multiple Sclerosis Signaling Pathway	1.34E+00
Upregulated ZT12	Pulmonary Healing Signaling Pathway	1.33E+00
Upregulated ZT12	Fc Epsilon RI Signaling	1.32E+00
Upregulated ZT12	Signaling by NOTCH3	1.32E+00
Upregulated ZT12	Activin Inhibin Signaling Pathway	1.30E+00

Table S3. Ingenuity Pathway Analysis on genes upregulated in BAMs at ZT0 versus ZT12.
All canonical pathways were identified by IPA analysis through the use of QIAGEN IPA (QIAGEN Inc.) on genes upregulated in BAMs at ZT0 versus ZT12. Input for IPA was DEGs identified by cell-level analysis. Output of IPA was filtered for $-\log_{10}$pvalue ≥ 1.3 and a minimum of 2 defining “molecules”.

bioRxiv preprint doi: https://doi.org/10.64898/2025.12.08.693074; this version posted December 11, 2025. The copyright holder for this preprint (which was not certified by peer review) is the author/funder, who has granted bioRxiv a license to display the preprint in perpetuity. It is made available under aCC-BY 4.0 International license.

Table S4

p_val	avg_log2FC	pct.1	pct.2	p_val_adj	gene
6.78E-63	3.94503927	0.136	0.011	1.57E-58	Plp1
1.14E-33	2.71367924	0.105	0.017	2.63E-29	Hspa1a
5.79E-14	1.47524523	0.089	0.031	1.34E-09	Cry1
3.38E-34	1.25546071	0.297	0.137	7.81E-30	Hsph1
5.91E-14	1.22006976	0.146	0.07	1.37E-09	Meg3
1.45E-36	1.1903588	0.366	0.207	3.36E-32	Hist1h2bc
1.18E-53	1.13421995	0.491	0.286	2.72E-49	Dynll1
6.01E-12	1.09470962	0.129	0.062	1.39E-07	Hist1h4d
6.14E-109	1.05134311	0.822	0.592	1.42E-104	Dnaja1
3.63E-72	1.02942423	0.814	0.623	8.40E-68	Gm26917
1.37E-14	0.84243593	0.195	0.105	3.16E-10	Cpeb4
6.81E-12	0.83366691	0.225	0.139	1.57E-07	Hist1h1e
5.11E-46	0.82785738	0.647	0.47	1.18E-41	Hsp90aa1
2.62E-38	0.79320295	0.593	0.41	6.06E-34	Chordc1
6.47E-12	0.79189411	0.184	0.103	1.50E-07	Gm15635
8.17E-14	0.78022472	0.278	0.176	1.89E-09	Kcnq1ot1
7.05E-10	0.7603613	0.236	0.155	1.63E-05	Slc15a3
2.40E-10	0.72893527	0.2	0.121	5.54E-06	Arntl
1.09E-13	0.71066866	0.371	0.264	2.52E-09	Thbd
1.50E-21	0.69285888	0.479	0.344	3.46E-17	Kitl
1.99E-35	0.69147849	0.782	0.654	4.61E-31	Ifi203
1.51E-15	0.67819529	0.388	0.265	3.48E-11	Ascc3
9.99E-09	0.6568647	0.293	0.211	0.00023092	Bcl2a1b
1.34E-14	0.63562404	0.415	0.294	3.10E-10	Trps1
5.93E-36	0.63080019	0.737	0.578	1.37E-31	Mat2a
1.18E-12	0.62968614	0.299	0.195	2.74E-08	Ahsa2
5.72E-11	0.61708059	0.416	0.334	1.32E-06	Cd52
3.71E-16	0.61576936	0.577	0.468	8.57E-12	Parp14
4.19E-08	0.60970305	0.232	0.158	0.00096876	Hivep1
2.33E-18	0.59633439	0.488	0.347	5.38E-14	Susd6
4.80E-139	0.58391064	0.996	0.982	1.11E-134	Hspa8
2.32E-21	0.57776715	0.584	0.443	5.36E-17	Atp2a2
7.03E-19	0.5686926	0.53	0.409	1.63E-14	mt-Nd3
1.43E-10	0.56785451	0.332	0.237	3.30E-06	Tmem170b
1.57E-16	0.56379636	0.528	0.403	3.63E-12	Tle4
1.29E-08	0.56329181	0.228	0.151	0.00029722	Banp
4.50E-24	0.54843024	0.709	0.574	1.04E-19	Runx1
1.75E-16	0.54784966	0.562	0.445	4.04E-12	Dleu2
1.72E-10	0.54775714	0.409	0.308	3.99E-06	Oas2
1.54E-11	0.54132355	0.417	0.32	3.55E-07	Stip1
1.36E-11	0.54084742	0.41	0.306	3.15E-07	Cdk12
1.01E-22	0.53765953	0.711	0.604	2.34E-18	Manf
2.34E-17	0.5373459	0.555	0.433	5.42E-13	Cacybp
7.09E-81	0.536593	1	0.997	1.64E-76	Malat1
3.16E-11	0.52747661	0.363	0.258	7.30E-07	Usp34
7.77E-12	0.52418537	0.407	0.297	1.80E-07	Zcchc7
6.43E-12	0.5233763	0.456	0.351	1.49E-07	Sgk3
6.33E-13	0.52133756	0.444	0.331	1.46E-08	Rbbp8
2.67E-31	0.52120504	0.782	0.654	6.18E-27	P4ha1
7.27E-07	0.5079181	0.292	0.22	0.01680893	Crim1
1.11E-07	0.50576406	0.323	0.246	0.00257467	Clock
1.94E-09	0.50299094	0.429	0.332	4.48E-05	Slc25a36
9.14E-08	0.50278209	0.34	0.257	0.00211259	Dok2
2.50E-09	0.4965903	0.405	0.317	5.77E-05	Krit1
6.34E-08	0.49202828	0.26	0.183	0.00146693	Tasor2
1.40E-07	0.47798225	0.328	0.247	0.00323408	Parp8
3.31E-07	0.47063699	0.198	0.135	0.00764557	Cemip2
4.55E-16	0.46743752	0.626	0.499	1.05E-11	Washc4
5.47E-13	0.46143944	0.534	0.417	1.27E-08	Agps
3.64E-19	0.46129109	0.703	0.59	8.42E-15	Itga4
5.57E-74	0.45926937	0.992	0.971	1.29E-69	Hsp90ab1
5.13E-41	0.45745298	1	0.999	1.19E-36	Gm42418
2.04E-08	0.4563238	0.433	0.344	0.00047177	Zc3h7a
1.05E-06	0.45597071	0.432	0.366	0.0241696	Cd93

bioRxiv preprint doi: https://doi.org/10.64898/2025.12.08.693074; this version posted December 11, 2025. The copyright holder for this preprint (which was not certified by peer review) is the author/funder, who has granted bioRxiv a license to display the preprint in perpetuity. It is made available under aCC-BY 4.0 International license.

8.27E-18	0.45353065	0.653	0.524	1.91E-13	Rab8b
2.94E-11	0.4522887	0.456	0.347	6.79E-07	Stag1
4.15E-09	0.4521747	0.282	0.199	9.59E-05	Hist1h1c
1.76E-09	0.45160714	0.38	0.282	4.07E-05	4932438A13Rik
6.65E-47	0.45085151	0.981	0.966	1.54E-42	Hspa5
6.97E-09	0.44926987	0.334	0.243	0.00016109	St3gal1
1.32E-06	0.44656291	0.303	0.229	0.03048111	Prpf39
2.40E-07	0.44603819	0.323	0.242	0.00554063	Trappc8
1.93E-12	0.44407784	0.58	0.471	4.45E-08	Erbin
4.57E-07	0.44312449	0.224	0.157	0.01057638	Med13
3.50E-16	0.44238319	0.613	0.477	8.10E-12	Ptpre
1.16E-12	0.43214752	0.571	0.457	2.69E-08	Ash1l
1.22E-17	0.42848751	0.703	0.58	2.83E-13	Ifi27l2a
7.11E-10	0.4283772	0.378	0.279	1.64E-05	Ktn1
9.75E-13	0.42547948	0.592	0.499	2.26E-08	Tmem33
2.74E-07	0.42322219	0.377	0.301	0.00632999	Matr3
1.99E-08	0.42296953	0.3	0.217	0.00046059	Nlrp3
3.89E-10	0.42253638	0.414	0.311	8.99E-06	Fam13b
3.33E-37	0.41708368	0.94	0.883	7.69E-33	Fcgr1
6.11E-11	0.41157586	0.537	0.435	1.41E-06	Pkn2
2.33E-18	0.41005454	0.79	0.694	5.38E-14	Mycbp2
1.10E-07	0.40523035	0.419	0.331	0.00253767	Golga4
2.53E-08	0.40471679	0.528	0.444	0.00058592	Luc7l3
1.58E-16	0.40464059	0.723	0.616	3.66E-12	Rock2
1.84E-19	0.40428967	0.81	0.711	4.26E-15	Calr
1.20E-22	0.40043558	0.89	0.829	2.77E-18	Abca1
9.35E-16	0.40021952	0.697	0.575	2.16E-11	Hnrnph1
1.39E-09	0.39961848	0.651	0.57	3.20E-05	Cx3cr1
2.97E-07	0.39573152	0.343	0.26	0.00687142	Herc2
5.38E-09	0.3933386	0.457	0.359	0.00012432	Ubn2
6.32E-09	0.3901873	0.562	0.477	0.00014615	Rreb1
1.54E-14	0.38963912	0.734	0.624	3.57E-10	Ccnd1
2.42E-22	0.38906428	0.864	0.794	5.59E-18	Ptprc
1.85E-08	0.38843976	0.555	0.469	0.00042882	Herc1
8.84E-52	0.38350266	0.996	0.995	2.05E-47	Rps29
1.63E-08	0.38225846	0.53	0.442	0.00037705	Phip
1.57E-08	0.37941048	0.519	0.431	0.00036365	Cep350
8.11E-10	0.37798118	0.579	0.479	1.88E-05	Nsd1
1.84E-18	0.37657989	0.771	0.653	4.25E-14	Cybc1
8.30E-11	0.37508309	0.611	0.515	1.92E-06	Tcea1
2.07E-07	0.37276273	0.399	0.313	0.00479018	Huwe1
1.97E-10	0.37121592	0.602	0.505	4.55E-06	Rnf141
9.56E-08	0.37099427	0.545	0.459	0.00221092	Spag9
1.72E-12	0.36592181	0.634	0.522	3.97E-08	Tra2a
2.72E-12	0.36567536	0.66	0.553	6.28E-08	Cflar
2.25E-10	0.36547448	0.631	0.527	5.21E-06	Slc8a1
1.78E-06	0.36498822	0.365	0.286	0.04111325	Atad2b
3.72E-09	0.36410683	0.513	0.413	8.60E-05	Tec
3.01E-07	0.36166488	0.346	0.264	0.00696831	Rbbp6
1.08E-06	0.36095557	0.191	0.135	0.02505779	Ttc39b
2.46E-08	0.35750708	0.506	0.411	0.00056895	Paxbp1
1.44E-09	0.35618013	0.535	0.432	3.34E-05	Ggta1
7.78E-10	0.35599419	0.571	0.47	1.80E-05	Dusp11
2.10E-12	0.35377065	0.657	0.544	4.86E-08	Macf1
4.32E-14	0.34626288	0.807	0.718	9.98E-10	Rbpj
3.04E-12	0.34574782	0.662	0.55	7.04E-08	Zswim6
9.64E-12	0.34561183	0.736	0.66	2.23E-07	Wasl
1.16E-06	0.34553122	0.484	0.402	0.02692171	Tlr8
1.47E-07	0.34482036	0.511	0.424	0.00339093	Fubp1
6.48E-08	0.34415811	0.568	0.493	0.00149922	Fkbp4
1.08E-06	0.34402786	0.219	0.159	0.02495716	Upf2
4.86E-07	0.3437435	0.488	0.406	0.01123153	Ppp1r9b
3.84E-07	0.34358117	0.563	0.478	0.00889028	9930111J21Rik2
1.71E-08	0.34147915	0.555	0.469	0.00039643	Csnk2a1
4.33E-09	0.3394796	0.626	0.536	0.00010015	Pcm1
5.14E-08	0.33852116	0.547	0.455	0.00118782	Raph1
8.67E-13	0.33398423	0.725	0.617	2.01E-08	Prrc2c

bioRxiv preprint doi: https://doi.org/10.64898/2025.12.08.693074; this version posted December 11, 2025. The copyright holder for this preprint (which was not certified by peer review) is the author/funder, who has granted bioRxiv a license to display the preprint in perpetuity. It is made available under aCC-BY 4.0 International license.

6.19E-09	0.33303083	0.616	0.527	0.00014319	Nktr
4.16E-07	0.33167084	0.491	0.404	0.00961366	Trip12
5.40E-12	0.33022698	0.765	0.685	1.25E-07	Top1
2.47E-11	0.32981211	0.709	0.639	5.71E-07	Cd2ap
5.83E-26	0.32943308	0.961	0.916	1.35E-21	Fyb
1.57E-19	0.32887471	0.93	0.887	3.64E-15	Lst1
8.27E-09	0.32813428	0.558	0.459	0.00019122	Atp8a1
4.56E-07	0.32765999	0.532	0.453	0.01054439	Hook3
5.15E-08	0.32763129	0.427	0.336	0.00119082	Nfat5
2.79E-09	0.32553863	0.632	0.565	6.46E-05	Hspe1
9.90E-09	0.32514284	0.552	0.454	0.00022884	Psd3
6.76E-29	0.32044958	0.985	0.973	1.56E-24	Rps28
7.18E-09	0.32020867	0.558	0.46	0.00016604	Kdm7a
4.40E-07	0.31101362	0.536	0.449	0.0101809	Birc6
3.19E-39	0.30960946	0.995	0.987	7.37E-35	Maf
2.17E-10	0.30953738	0.708	0.617	5.02E-06	Syk
5.60E-12	0.30923724	0.795	0.728	1.30E-07	Sec62
5.22E-13	0.30910665	0.838	0.762	1.21E-08	Dusp6
9.90E-09	0.30753971	0.554	0.454	0.00022882	Dock10
3.47E-08	0.30534226	0.669	0.592	0.00080157	Wls
7.08E-09	0.30520003	0.479	0.38	0.00016374	Gls
8.29E-27	0.30514424	0.971	0.958	1.92E-22	Mef2c
4.70E-07	0.30164176	0.452	0.365	0.01087123	Plekho2
4.95E-10	0.30162946	0.695	0.599	1.14E-05	Tlr4
2.28E-15	0.30051345	0.875	0.842	5.28E-11	Atp6v0a1
5.63E-07	0.3003426	0.373	0.293	0.01300963	Stt3b
2.66E-09	0.29866068	0.7	0.621	6.15E-05	Ywhah
2.68E-11	0.29822625	0.674	0.566	6.20E-07	Atrx
1.51E-06	0.29684643	0.303	0.233	0.03489259	Tmem164
4.72E-08	0.2950674	0.685	0.62	0.00109208	Sash1
4.04E-17	0.29463244	0.909	0.849	9.35E-13	Itsn1
2.62E-19	0.29307872	0.958	0.919	6.07E-15	Zeb2
2.87E-18	0.29050114	0.914	0.859	6.63E-14	Mbnl1
2.86E-11	0.28906419	0.734	0.637	6.62E-07	Wnk1
5.59E-08	0.28825607	0.616	0.527	0.00129342	Wdfy2
6.23E-08	0.28687925	0.549	0.455	0.00144092	Ccnt2
2.76E-08	0.28658252	0.706	0.63	0.00063709	Fndc3a
4.44E-07	0.28591405	0.623	0.546	0.01026368	Tut4
2.39E-09	0.28207197	0.766	0.7	5.53E-05	Dock8
1.83E-07	0.28073581	0.595	0.505	0.00424035	Ep300
9.73E-09	0.27964626	0.675	0.582	0.00022499	Ptges3
4.29E-07	0.2785973	0.65	0.575	0.00992121	Rbm5
1.25E-23	0.27850356	0.979	0.962	2.90E-19	Rps27
2.21E-08	0.27767004	0.681	0.593	0.00051127	Aftph
1.38E-08	0.27493037	0.73	0.66	0.00031828	Srsf11
6.18E-24	0.27460657	0.982	0.971	1.43E-19	Hsp90b1
1.52E-12	0.27456593	0.859	0.791	3.50E-08	Kctd12
1.48E-25	0.27453743	0.977	0.961	3.42E-21	Rbm39
1.59E-08	0.27395773	0.737	0.67	0.00036712	Sfpq
5.36E-27	0.27124567	0.99	0.981	1.24E-22	Marcks
2.30E-08	0.26800762	0.661	0.572	0.00053117	Nrip1
2.28E-10	0.26740484	0.882	0.845	5.28E-06	Ssh2
1.55E-06	0.26539399	0.583	0.5	0.03586221	Dnajc13
2.11E-18	0.2618708	0.96	0.918	4.88E-14	Son
5.67E-07	0.26078278	0.697	0.646	0.01311538	Sdf2l1
1.81E-06	0.26071978	0.483	0.4	0.04183474	Zfc3h1
2.98E-10	0.25755303	0.798	0.723	6.89E-06	Pafah1b1
1.32E-06	0.25701169	0.704	0.636	0.03059234	Ogt
1.06E-12	0.25589979	0.903	0.845	2.44E-08	Clec2d
1.98E-07	0.25357322	0.745	0.677	0.00456785	Celf2
4.02E-14	0.25137942	0.92	0.889	9.29E-10	Slco2b1
7.56E-13	0.24613303	0.926	0.913	1.75E-08	mt-Nd4
4.01E-20	0.24589521	0.989	0.984	9.26E-16	Rps21
3.62E-16	0.24444716	0.98	0.971	8.38E-12	Rpl39
4.08E-22	0.24322081	0.992	0.989	9.43E-18	Rpl37a
1.28E-15	0.24076386	0.941	0.916	2.96E-11	Abca9
1.94E-14	0.24012431	0.937	0.891	4.48E-10	Lrrc25

bioRxiv preprint doi: https://doi.org/10.64898/2025.12.08.693074; this version posted December 11, 2025. The copyright holder for this preprint (which was not certified by peer review) is the author/funder, who has granted bioRxiv a license to display the preprint in perpetuity. It is made available under aCC-BY 4.0 International license.

1.46E-07	0.24009457	0.76	0.687	0.00338664	Lgals3bp
2.54E-07	0.23829648	0.802	0.734	0.00587101	Pak2
1.58E-08	0.23751068	0.826	0.769	0.00036621	Eif4g2
8.00E-10	0.23750094	0.842	0.775	1.85E-05	Xbp1
1.67E-06	0.23726229	0.699	0.627	0.03869419	Morc3
4.22E-08	0.23681176	0.8	0.732	0.00097564	Bmp2k
2.81E-07	0.23373678	0.786	0.717	0.0065053	Slfn2
1.54E-06	0.23235832	0.687	0.611	0.03556693	Tnks2
1.29E-07	0.23049204	0.8	0.755	0.00297263	Ccdc50
1.65E-13	0.22661397	0.971	0.963	3.81E-09	Nrros
2.46E-08	0.22447456	0.923	0.906	0.00056872	AC149090.1
1.42E-08	0.22235104	0.872	0.831	0.00032943	Ptbp3
2.14E-06	0.22202882	0.393	0.318	0.04943964	Ccdc93
2.06E-07	0.21665945	0.823	0.764	0.00476678	Stk17b
6.32E-10	0.21415032	0.943	0.917	1.46E-05	Ifitm3
5.40E-17	0.21349476	0.997	0.993	1.25E-12	B2m
2.00E-06	0.21197717	0.755	0.72	0.046323	Ubap2l
2.70E-07	0.2080027	0.863	0.833	0.00624751	Itgb1
4.72E-11	0.20252323	0.953	0.923	1.09E-06	Cd164
8.73E-07	0.19782285	0.566	0.481	0.02017923	Rock1
5.05E-08	0.19648467	0.891	0.846	0.00116884	Tm6sf1
1.28E-06	0.19598547	0.832	0.783	0.02949145	Tcf4
1.81E-06	0.19250614	0.829	0.783	0.04177412	Dclre1c
2.09E-08	0.1834713	0.947	0.919	0.00048385	Rrbp1
1.32E-15	0.18317579	0.996	0.997	3.04E-11	Dab2
4.70E-14	0.17907623	0.998	1	1.09E-09	Mrc1
2.18E-08	0.16909692	0.933	0.89	0.00050511	C5ar1
1.84E-06	0.16785828	0.514	0.436	0.04244757	Rbm26
5.12E-09	0.16411775	0.969	0.946	0.00011838	Srrm2
1.17E-06	0.15871106	0.792	0.719	0.02716863	Plxnb2

Table S4. DEGs upregulated at ZT0 in BAMs from cell-level analysis.
All genes upregulated at ZT0 from cell-level analysis of BAMs across all four time points. Gene list is filtered for adjusted p-value <0.05. Cell-level DEG analysis approach is described in **Methods**.

bioRxiv preprint doi: https://doi.org/10.64898/2025.12.08.693074; this version posted December 11, 2025. The copyright holder for this preprint (which was not certified by peer review) is the author/funder, who has granted bioRxiv a license to display the preprint in perpetuity. It is made available under aCC-BY 4.0 International license.

Table S5:

gene	p_val	avg_log2FC	pct.1	pct.2	p_val_adj
H2-DMa	7.45E-131	2.032479259	1	1	1.73E-126
H2-DMb1	9.17E-76	1.856490689	1	1	2.13E-71
H2-Eb1	2.05E-70	4.257982139	1	1	4.75E-66
Ly9	6.39E-63	1.617412331	1	1	1.48E-58
H2-Aa	1.00E-56	3.867580065	1	1	2.33E-52
Afdn	2.83E-55	1.802312325	1	1	6.57E-51
Gm21188	3.78E-55	2.163801399	1	1	8.78E-51
Klra2	1.76E-54	1.844353574	1	1	4.07E-50
Cyp4f18	2.48E-54	1.813618808	1	1	5.76E-50
Lat2	6.30E-54	1.939466708	1	1	1.46E-49
Lair1	2.80E-51	1.129861449	1	1	6.49E-47
Gngt2	1.03E-48	1.724861946	1	1	2.38E-44
Abi3	1.79E-48	1.797124646	1	1	4.15E-44
Fgl2	1.19E-46	1.472512712	1	1	2.76E-42
Fxyd5	2.39E-43	1.930730024	1	1	5.55E-39
Axl	6.81E-43	1.708323519	1	1	1.58E-38
Havcr2	2.08E-42	1.637603327	1	1	4.84E-38
Lpcat2	9.69E-42	1.174644712	1	1	2.25E-37
Runx3	2.67E-41	1.617183669	1	1	6.20E-37
Ccl8	1.04E-40	2.053385741	1	1	2.41E-36
Tmem119	1.50E-39	1.630866398	1	1	3.48E-35
F11r	1.13E-38	1.484056048	1	1	2.62E-34
Mmp14	1.54E-38	1.891335065	1	1	3.57E-34
H2-Ab1	7.69E-38	3.530336296	1	1	1.78E-33
Mpeg1	2.17E-37	1.253970605	1	1	5.02E-33
A530040E14Rik	3.75E-37	1.477525556	1	1	8.69E-33
Xdh	8.87E-37	1.498200349	1	1	2.06E-32
Olfml3	1.48E-36	1.816560059	1	1	3.43E-32
Slamf7	1.53E-36	1.871096307	1	0.667	3.54E-32
Cxcl16	1.76E-36	1.14256653	1	1	4.09E-32
Cd72	3.23E-35	1.727236243	1	1	7.50E-31
Fcgrt	4.27E-34	-0.884111487	1	1	9.89E-30
Cd74	8.29E-34	3.523797668	1	1	1.92E-29
Tjp1	5.76E-33	1.543749576	1	1	1.34E-28
AW112010	7.80E-32	1.375080706	1	1	1.81E-27
Acvrl1	3.90E-31	1.30916705	1	1	9.04E-27
Ednrb	5.01E-31	-1.694202983	1	1	1.16E-26
Slc40a1	5.79E-31	-1.236578156	1	1	1.34E-26
H2-Q4	3.55E-30	1.000922566	1	1	8.23E-26
Plbd1	1.58E-29	2.399325571	1	0.833	3.66E-25
Ms4a4c	5.06E-28	1.591712899	1	1	1.17E-23
Tnfsf13	1.49E-27	1.133106159	1	1	3.46E-23
H2-Q7	2.88E-27	1.402849016	1	1	6.67E-23
Cd81	1.25E-26	1.157729325	1	1	2.89E-22
Pmepa1	1.32E-26	1.423776367	1	1	3.07E-22
Sp100	1.41E-26	0.915385454	1	1	3.28E-22
Dab2	2.26E-26	-0.774222404	1	1	5.25E-22
Cd200r4	1.06E-25	1.345380785	1	1	2.46E-21
Ctss	1.28E-25	0.789924233	1	1	2.96E-21
Slamf8	1.28E-25	1.333798825	1	0.833	2.97E-21
Il10ra	1.76E-25	0.724987204	1	1	4.07E-21
C2	1.06E-24	-0.922343375	1	1	2.45E-20
Cxcl13	2.98E-24	3.447879795	1	0	6.92E-20
Gm36161	3.31E-24	2.455447686	1	1	7.67E-20
Grk3	4.48E-23	1.270959588	1	1	1.04E-18
Mgst1	9.56E-23	1.335960682	1	1	2.22E-18
Bcl2a1a	1.60E-22	1.246522706	1	1	3.72E-18
Susd3	6.99E-22	0.976118564	1	1	1.62E-17
H2-K1	8.78E-22	0.831134646	1	1	2.04E-17
Cd28	3.04E-21	-1.252244212	1	1	7.05E-17
Clec10a	5.08E-21	-1.124389656	1	1	1.18E-16
Mmp2	5.36E-21	1.276616218	1	1	1.24E-16
Tor3a	8.46E-21	1.035635226	1	1	1.96E-16
Slc41a2	8.57E-21	1.187539597	1	0.833	1.99E-16

bioRxiv preprint doi: https://doi.org/10.64898/2025.12.08.693074; this version posted December 11, 2025. The copyright holder for this preprint (which was not certified by peer review) is the author/funder, who has granted bioRxiv a license to display the preprint in perpetuity. It is made available under aCC-BY 4.0 International license.

Scimp	1.93E-20	1.121815121	1	1	4.47E-16
H2-M3	2.10E-20	0.77600683	1	1	4.86E-16
Atp1a3	2.36E-20	1.179808024	1	1	5.47E-16
Coro1a	2.42E-20	1.183462158	1	1	5.61E-16
Cd52	8.77E-20	1.858342671	1	1	2.03E-15
Glipr1	9.65E-20	1.184789685	1	1	2.24E-15
Plxdc1	1.06E-19	1.182852203	1	1	2.45E-15
Gm15964	1.06E-19	1.208041128	1	1	2.45E-15
Cd209c	2.29E-19	1.179846646	1	1	5.31E-15
Pirb	3.46E-19	0.84407445	1	1	8.03E-15
Bcl2a1b	4.19E-19	1.240121257	1	1	9.71E-15
Ptprk	6.44E-19	-1.308790433	1	1	1.49E-14
Tagap	8.02E-19	0.997198601	1	1	1.86E-14
Cxcl14	1.24E-18	1.259133487	1	0.833	2.87E-14
Junb	1.72E-18	1.189830364	1	1	3.98E-14
H2-D1	4.99E-18	0.851474917	1	1	1.16E-13
Tmem176a	1.03E-17	0.812012999	1	1	2.39E-13
App	1.13E-17	-0.653956197	1	1	2.63E-13
Tnfsf13b	1.67E-17	1.183890361	1	1	3.88E-13
Crybg1	1.83E-17	1.087718855	1	0.833	4.24E-13
Hexb	2.17E-17	1.125744301	1	1	5.03E-13
Tspan32	2.85E-17	1.16346744	1	0.667	6.61E-13
Gimap6	2.95E-17	-1.092061008	0.75	1	6.83E-13
Cpq	3.83E-17	0.829771343	1	1	8.89E-13
Il2rg	5.87E-17	2.157097603	1	1	1.36E-12
Rhob	8.67E-17	0.818843882	1	1	2.01E-12
H2-T22	9.54E-17	0.880841555	1	1	2.21E-12
Igfbp4	1.12E-16	-0.935910139	1	1	2.59E-12
Clec1a	1.57E-16	1.110318557	1	0.667	3.63E-12
Sdc3	1.70E-16	0.810818662	1	1	3.95E-12
Cd209a	2.02E-16	1.138904332	1	1	4.69E-12
Mcub	2.39E-16	1.085477441	1	1	5.54E-12
Cd93	2.54E-16	0.96812386	1	1	5.88E-12
St8sia6	2.98E-16	1.118910827	1	0.833	6.92E-12
Sp140	3.06E-16	0.829813013	1	1	7.09E-12
Ifi44	3.40E-16	1.220025846	1	0.833	7.89E-12
Basp1	3.41E-16	0.847588178	1	1	7.92E-12
Tbc1d4	6.57E-16	-0.893128778	1	1	1.52E-11
Lilra5	7.29E-16	0.929718023	1	1	1.69E-11
Vcam1	7.39E-16	1.228955351	1	1	1.71E-11
Slc11a1	1.57E-15	0.588042475	1	1	3.65E-11
Cd22	2.15E-15	1.122453843	1	1	5.00E-11
Lrp6	2.80E-15	-0.764157847	1	1	6.49E-11
Colec12	3.00E-15	-1.169850788	1	1	6.96E-11
Gstp1	3.83E-15	-0.849465815	1	1	8.88E-11
Gpr65	4.86E-15	0.851158142	1	1	1.13E-10
Dst	7.62E-15	1.040032522	1	1	1.77E-10
Klf2	1.32E-14	1.882300368	1	1	3.05E-10
Plk3	1.57E-14	1.015636305	1	1	3.64E-10
Abca1	2.58E-14	-0.955930307	1	1	5.99E-10
Psmb8	2.90E-14	0.589640213	1	1	6.72E-10
Padi2	2.94E-14	0.994917995	1	1	6.82E-10
Oasl2	2.98E-14	0.922191529	1	1	6.91E-10
Cfh	3.00E-14	-0.660597226	1	1	6.96E-10
Ldlrad3	3.46E-14	1.076142943	1	0.833	8.02E-10
H2-Q6	3.57E-14	1.647508849	1	1	8.27E-10
Stab1	5.17E-14	-0.733289034	1	1	1.20E-09
Cd9	5.33E-14	1.58342125	1	1	1.24E-09
Cpeb1	5.47E-14	1.083767944	1	1	1.27E-09
Tcirg1	6.28E-14	0.706567212	1	1	1.46E-09
Clec12a	6.54E-14	0.983204814	1	1	1.52E-09
Klrk1	6.56E-14	1.149537504	1	0.333	1.52E-09
Rasal3	7.22E-14	1.002149643	1	1	1.67E-09
AY036118	1.67E-13	-1.011947951	1	1	3.88E-09
Ifi209	1.78E-13	0.954385448	1	1	4.13E-09
Phf11b	1.87E-13	1.11264362	1	1	4.34E-09
Tmem51	1.97E-13	1.017501379	1	1	4.56E-09

bioRxiv preprint doi: https://doi.org/10.64898/2025.12.08.693074; this version posted December 11, 2025. The copyright holder for this preprint (which was not certified by peer review) is the author/funder, who has granted bioRxiv a license to display the preprint in perpetuity. It is made available under aCC-BY 4.0 International license.

Ptk2b	2.53E-13	1.016291329	1	1	5.88E-09
Cd4	2.96E-13	-0.794016516	1	1	6.87E-09
Gm35154	3.14E-13	1.050422559	1	1	7.29E-09
Clec2i	3.84E-13	0.979581982	1	1	8.90E-09
Zmynd15	4.29E-13	1.125521412	1	1	9.94E-09
Gprc5c	4.84E-13	-0.81646769	0.75	1	1.12E-08
Fcgr4	6.78E-13	0.990678644	1	1	1.57E-08
Skil	7.45E-13	0.894185274	1	1	1.73E-08
Egfr	7.62E-13	-0.918469734	0.75	1	1.77E-08
Batf3	1.27E-12	1.04679157	1	1	2.95E-08
Tmem50b	1.28E-12	0.726341052	1	1	2.97E-08
Myc	1.39E-12	0.975693554	1	1	3.21E-08
Chic1	1.47E-12	0.976299992	1	0.5	3.41E-08
Tap1	1.54E-12	0.687293502	1	1	3.57E-08
Dtx3	1.63E-12	0.837584653	1	1	3.77E-08
Crlf2	1.77E-12	0.63116524	1	1	4.11E-08
Plxnc1	2.25E-12	-0.66138976	1	1	5.21E-08
Phf11a	2.27E-12	1.051709353	1	1	5.26E-08
Nedd9	2.36E-12	0.964441163	1	1	5.48E-08
Gabbr1	2.46E-12	-0.818564509	1	1	5.71E-08
A530032D15Rik	2.81E-12	0.967361063	1	1	6.51E-08
S1pr1	3.35E-12	-0.648615721	1	1	7.77E-08
C4b	4.47E-12	0.670919511	1	1	1.04E-07
Gm35853	6.00E-12	0.967706187	1	1	1.39E-07
Tle1	7.11E-12	0.962655829	1	1	1.65E-07
Ppcdc	8.40E-12	0.932777433	1	1	1.95E-07
AC168977.2	1.22E-11	0.949701478	1	0.833	2.83E-07
Smad7	1.45E-11	1.002699444	1	1	3.36E-07
Tent5c	1.55E-11	1.000484308	1	1	3.60E-07
Ifi27l2a	1.71E-11	0.947369047	1	1	3.97E-07
Ciita	1.90E-11	1.122654267	1	1	4.41E-07
Ap1b1	2.29E-11	-0.6322397	1	1	5.31E-07
Selenom	2.31E-11	0.838127587	1	1	5.35E-07
Tspan7	2.53E-11	-0.835345184	1	1	5.88E-07
Pvt1	3.16E-11	0.946649556	1	1	7.32E-07
Ppp1r9a	3.24E-11	-0.60386298	1	1	7.51E-07
Mctp1	3.29E-11	-0.60227237	1	1	7.62E-07
Smim1	3.44E-11	-0.696109026	1	1	7.99E-07
Prps2	3.45E-11	-0.670719376	1	1	8.01E-07
Mrc1	3.55E-11	-0.638030567	1	1	8.24E-07
C1qtnf1	4.07E-11	0.986106591	1	1	9.44E-07
Higd2a	4.35E-11	-0.691058966	1	1	1.01E-06
Cfp	4.57E-11	-0.772636458	1	1	1.06E-06
Gpr132	4.79E-11	0.943244408	1	0.5	1.11E-06
Ccl6	5.01E-11	0.856727665	1	1	1.16E-06
Trerf1	7.57E-11	0.94727635	1	1	1.76E-06
Hvcn1	8.15E-11	0.831869118	1	1	1.89E-06
Cd209d	8.42E-11	1.605245002	1	1	1.95E-06
Rad51b	1.03E-10	0.973403809	1	1	2.39E-06
Abcg2	1.05E-10	1.082374993	1	1	2.44E-06
Aplp2	1.35E-10	-0.594418206	1	1	3.13E-06
Got1	1.48E-10	0.767412171	1	1	3.43E-06
Rgs7bp	1.48E-10	-0.649548858	1	1	3.44E-06
Tmem176b	1.53E-10	0.598056406	1	1	3.55E-06
Clic4	1.56E-10	0.846040354	1	1	3.63E-06
Pf4	2.40E-10	-0.682428537	1	1	5.57E-06
Sgk1	2.49E-10	0.977039037	1	1	5.78E-06
Dclre1c	3.05E-10	-0.613381249	1	1	7.07E-06
Cd200r1	3.18E-10	-0.595006657	1	1	7.37E-06
Reps2	3.35E-10	-0.617586803	1	1	7.76E-06
Akt3	3.49E-10	0.902281975	1	1	8.08E-06
Bcl2a1d	4.10E-10	0.960419643	1	0.833	9.51E-06
Eif4e3	5.27E-10	0.822922768	1	1	1.22E-05
Oasl1	6.22E-10	0.970551009	1	1	1.44E-05
Gdpd3	6.33E-10	0.961017138	1	1	1.47E-05
Sft2d2	7.52E-10	-0.581324835	1	1	1.74E-05
AC133103.1	7.63E-10	0.931664984	0.75	0.333	1.77E-05

bioRxiv preprint doi: https://doi.org/10.64898/2025.12.08.693074; this version posted December 11, 2025. The copyright holder for this preprint (which was not certified by peer review) is the author/funder, who has granted bioRxiv a license to display the preprint in perpetuity. It is made available under aCC-BY 4.0 International license.

Sirpb1b	8.18E-10	0.937063457	1	0.667	1.90E-05
Mlxipl	9.27E-10	0.980292685	1	1	2.15E-05
Col14a1	1.23E-09	1.086624659	1	1	2.86E-05
Slc12a9	1.32E-09	0.787452073	1	1	3.06E-05
Tnfaip2	1.55E-09	0.995671206	1	1	3.59E-05
Rasal2	1.62E-09	-0.701083436	1	1	3.76E-05
Lsp1	2.00E-09	1.004393518	1	1	4.64E-05
Arhgap27	2.45E-09	1.097958817	1	1	5.68E-05
C3	3.40E-09	1.616139005	1	0.833	7.89E-05
Sh3pxd2a	3.84E-09	-0.637799745	1	1	8.91E-05
Chst12	4.02E-09	0.726002412	1	1	9.33E-05
Lst1	4.27E-09	-0.665871066	1	1	9.91E-05
C130026I21Rik	6.89E-09	0.913893455	1	1	0.000159875
Arl5c	7.06E-09	0.99464524	1	1	0.000163775
Ccr5	7.58E-09	1.43988084	1	1	0.000175873
Hpse	7.79E-09	0.963298624	1	1	0.000180594
Cd83	8.04E-09	0.840714291	1	1	0.000186382
Ifi213	8.18E-09	1.223016496	1	1	0.000189635
Ramp1	9.79E-09	0.630753862	1	1	0.000226984
Slc1a5	1.00E-08	0.864571375	1	1	0.000232254
Gpr18	1.30E-08	0.848766223	1	0.667	0.000301404
Irf1	1.50E-08	0.675320981	1	1	0.000347309
Csprs	1.61E-08	0.881218114	1	1	0.000373873
Cers4	1.67E-08	0.92012499	1	1	0.000388033
Ncf4	1.90E-08	0.669523447	1	1	0.00044101
Tlr12	2.32E-08	0.895608653	1	1	0.000537923
Gpr165	2.38E-08	-0.731660654	1	1	0.000553054
Agpat4	2.46E-08	0.883043967	1	1	0.000569784
Pfkfb3	2.86E-08	0.832373501	1	1	0.000664096
Thbd	2.89E-08	-0.984956575	1	1	0.000670791
Rcbtb2	3.30E-08	0.650179738	1	1	0.000765107
Mafb	3.54E-08	1.36139607	1	1	0.000821667
Iigp1	3.62E-08	1.239097936	1	1	0.000839736
Prr5l	3.63E-08	0.929887535	1	1	0.00084203
Cdc42ep3	3.82E-08	0.930387162	0.75	0.667	0.000886054
Icam1	3.83E-08	0.938788772	1	1	0.000888674
Mageh1	4.19E-08	0.947768694	1	1	0.000972085
Dnaaf3	4.26E-08	0.823697636	1	1	0.000987254
Trp53i11	4.29E-08	0.872213994	1	1	0.000994869
Pira2	4.83E-08	0.846130427	1	0.5	0.001119395
Ccdc102a	5.69E-08	0.819679831	1	0.667	0.001320569
Mical1	5.79E-08	0.85434827	1	1	0.001343728
Tslp	6.26E-08	-1.153818012	1	1	0.001452494
Ccnd2	7.11E-08	1.379816236	1	1	0.001649612
Sorl1	7.25E-08	1.230122487	1	1	0.001682382
Bin2	7.66E-08	0.635129113	1	1	0.001776794
Usp2	7.71E-08	0.794922592	1	1	0.001787802
Lgals3	8.85E-08	1.361062543	1	1	0.002053041
Lyz1	9.35E-08	1.370276132	1	0.667	0.002168325
Tspan13	1.00E-07	1.009755193	1	1	0.002323395
Kif1a	1.03E-07	0.874714275	1	1	0.00239979
Rab11fip1	1.06E-07	0.804997643	0.75	0.333	0.002457984
Atf3	1.16E-07	0.990183028	1	1	0.00268093
Vasp	1.16E-07	0.600143294	1	1	0.002681344
Ptprm	1.17E-07	0.983346468	1	1	0.002721204
Sirpb1c	1.20E-07	0.785926305	1	0.333	0.002777572
Gm20559	1.25E-07	0.921929133	1	1	0.002902239
Siglece	1.33E-07	0.743173785	1	1	0.003085986
Gdf3	1.41E-07	1.376069511	1	0.833	0.003267918
Gstt1	1.43E-07	0.84996237	1	0.833	0.003308219
Emilin1	1.49E-07	0.87307672	1	1	0.003444293
Stat1	1.55E-07	0.733123543	1	1	0.003600634
Tank	1.76E-07	0.743272358	1	1	0.004079436
Asb13	1.86E-07	0.720432488	1	1	0.004308583
Parvg	1.96E-07	0.866511391	1	1	0.004534793
Lars2	2.10E-07	-0.925138246	1	1	0.004877717
Itsn1	2.47E-07	-0.757047144	1	1	0.005723618

bioRxiv preprint doi: https://doi.org/10.64898/2025.12.08.693074; this version posted December 11, 2025. The copyright holder for this preprint (which was not certified by peer review) is the author/funder, who has granted bioRxiv a license to display the preprint in perpetuity. It is made available under aCC-BY 4.0 International license.

Stx3	2.77E-07	0.849017056	1	0.833	0.006429312
Ptger4	2.82E-07	0.894983109	1	1	0.006547564
Nuak2	2.85E-07	0.774978769	1	1	0.00661773
Clcn5	2.88E-07	-0.975790175	1	1	0.006683453
Clec4b1	3.29E-07	0.861604216	1	1	0.007619406
Naalad2	3.48E-07	0.81840525	1	1	0.008061616
Raph1	4.37E-07	-0.584358306	1	1	0.01013032
Ptn	5.06E-07	0.883447222	1	1	0.011725243
Emilin2	5.55E-07	0.797792242	1	1	0.012869672
Socs3	5.74E-07	0.824848471	1	1	0.013323153
Il1b	5.84E-07	1.723180483	1	1	0.013551441
Isg20	5.90E-07	0.863073515	1	1	0.013677305
Irf7	6.67E-07	1.533107008	1	1	0.015458609
Dok2	6.77E-07	-0.637608407	1	1	0.015697626
Zfp691	7.93E-07	0.879032329	1	1	0.018396472
Zbp1	7.97E-07	0.855311301	1	1	0.018473328
AU020206	8.09E-07	0.747130571	1	1	0.018757161
Rgs1	8.53E-07	0.829525919	1	0.833	0.019789926
Ccdc86	8.55E-07	0.698857942	1	1	0.019819548
Timp3	1.21E-06	0.83017052	0.5	0.667	0.027997657
Dnajc25	1.24E-06	0.779076096	1	1	0.028686661
Fut4	1.35E-06	0.844480521	1	1	0.031291143
Bcl3	1.53E-06	0.830799671	1	1	0.035439723
Cd164	2.14E-06	-0.726135072	1	1	0.049531903

Table S5. Adult versus aged BAM DEGs from pseudobulk comparison. Note: Positive fold changes represent upregulation in aged BAMs.

bioRxiv preprint doi: https://doi.org/10.64898/2025.12.08.693074; this version posted December 11, 2025. The copyright holder for this preprint (which was not certified by peer review) is the author/funder, who has granted bioRxiv a license to display the preprint in perpetuity. It is made available under aCC-BY 4.0 International license.

Table S6:

Antigen	Fluorophore	Clone	Manufacturer	Cat number	Dilution	Note
CD11b	AF488	M1/70	BioLegend	101217	200	
CD11b	BUV395	M1/70	BD Biosciences	563553	200	
CD11b	BV510	M1/70	BioLegend	101245	200	
CD11b	BV605	M1/70	BioLegend	101237	200	
CD11c	PE-Cy5.5	N418	Thermo Fisher Scientific	35-0114-82	200	
CD206	BV421	C068C2	BioLegend	141717	200	
CD206	PE	C068C2	BioLegend	141720	100	
CD38	PE	90	BioLegend	102708	200	
CD38	PE-Cy7	90	BioLegend	102718	200	
CD38	BV421	90	BioLegend	102732	200	
CD38	BUV395	90/CD38	BD Biosciences	740245	200	
CD38	BUV805	90/CD38	BD Biosciences	741955	200	
CD45	PE-Cy5.5	30-F11	Thermo Fisher Scientific	35-0451-82	500	Used 1:200 for FEAST
CD45	PE	30-F11	BioLegend	103106	500	
CD45	BUV805	30-F11	BD Biosciences	568336	200	
CD64	PE-Cy7	X54-5/7.1	BioLegend	139313	100	
CD64	BV421	X54-5/7.1	BioLegend	139309	100	
CD68	APC-Cy7	FA-11	BioLegend	137024	400	
CX3CR1	BV711	SA011F11	BioLegend	149031	800	
CX3CR1	AF488	SA011F11	BioLegend	149022	800	
CX3CR1	AF647	SA011F11	BioLegend	149004	800	
GR-1	BUV805	RB6-8C5	BD Biosciences	741920	100	
GR-1	AF488	RB6-8C5	BioLegend	108417	100	
GR-1	PE	RB6-8C5	BioLegend	108408	100	
Ly-6C	BV510	HK1.4	BioLegend	128033	200	
MHC-II	BV785	M5/114.15.2	BioLegend	107645	200	
β-Amyloid, 1-16	AF647	6E10	BioLegend	803021	200	

Table S6. Antibodies used for flow cytometry applications.
Note: Panels and concentrations were adjusted for the fixed cell FEAST preparation.
